# Ectomycorrhizal fungi recruit hyphae-associated bacteria that metabolize thiamine to promote pine symbiosis

**DOI:** 10.1093/ismejo/wraf290

**Published:** 2025-12-27

**Authors:** Jiale Zhu, Mengya Yu, Tingyu Zheng, Jie Zhang, Genyue Cao, Xiaohan Wu, Chuanchao Dai, Yaseen Ullah, Wei Zhang, Yong Jia

**Affiliations:** Jiangsu Key Laboratory of Pathogenesis and Ecosystems, Jiangsu Engineering and Technology Research Center for Industrialization of Microbial Resources, College of Life Sciences, Nanjing Normal University, No. 1 Wenyuan Road, Qixia District, Nanjing, Jiangsu 210023, China; Jiangsu Key Laboratory of Pathogenesis and Ecosystems, Jiangsu Engineering and Technology Research Center for Industrialization of Microbial Resources, College of Life Sciences, Nanjing Normal University, No. 1 Wenyuan Road, Qixia District, Nanjing, Jiangsu 210023, China; Jiangsu Key Laboratory of Pathogenesis and Ecosystems, Jiangsu Engineering and Technology Research Center for Industrialization of Microbial Resources, College of Life Sciences, Nanjing Normal University, No. 1 Wenyuan Road, Qixia District, Nanjing, Jiangsu 210023, China; Jiangsu Key Laboratory of Pathogenesis and Ecosystems, Jiangsu Engineering and Technology Research Center for Industrialization of Microbial Resources, College of Life Sciences, Nanjing Normal University, No. 1 Wenyuan Road, Qixia District, Nanjing, Jiangsu 210023, China; Jiangsu Key Laboratory of Pathogenesis and Ecosystems, Jiangsu Engineering and Technology Research Center for Industrialization of Microbial Resources, College of Life Sciences, Nanjing Normal University, No. 1 Wenyuan Road, Qixia District, Nanjing, Jiangsu 210023, China; Jiangsu Key Laboratory of Pathogenesis and Ecosystems, Jiangsu Engineering and Technology Research Center for Industrialization of Microbial Resources, College of Life Sciences, Nanjing Normal University, No. 1 Wenyuan Road, Qixia District, Nanjing, Jiangsu 210023, China; Jiangsu Key Laboratory of Pathogenesis and Ecosystems, Jiangsu Engineering and Technology Research Center for Industrialization of Microbial Resources, College of Life Sciences, Nanjing Normal University, No. 1 Wenyuan Road, Qixia District, Nanjing, Jiangsu 210023, China; Jiangsu Key Laboratory of Pathogenesis and Ecosystems, Jiangsu Engineering and Technology Research Center for Industrialization of Microbial Resources, College of Life Sciences, Nanjing Normal University, No. 1 Wenyuan Road, Qixia District, Nanjing, Jiangsu 210023, China; Jiangsu Key Laboratory of Pathogenesis and Ecosystems, Jiangsu Engineering and Technology Research Center for Industrialization of Microbial Resources, College of Life Sciences, Nanjing Normal University, No. 1 Wenyuan Road, Qixia District, Nanjing, Jiangsu 210023, China; Jiangsu Key Laboratory of Pathogenesis and Ecosystems, Jiangsu Engineering and Technology Research Center for Industrialization of Microbial Resources, College of Life Sciences, Nanjing Normal University, No. 1 Wenyuan Road, Qixia District, Nanjing, Jiangsu 210023, China

**Keywords:** ectomycorrhizal fungi, hyphae-associated bacterium, thiamine, synergistic mechanisms

## Abstract

Ectomycorrhizal fungi form symbiotic relationships with a wide range of terrestrial plants, acquiring carbohydrates for themselves and promoting nutrient uptake in their host plants. However, some ectomycorrhizal fungi cannot effectively obtain the thiamine necessary for growth from their host or synthesize it themselves. Ectomycorrhizal fungi can recruit hypha-associated microorganisms, which play a vital role in promoting nutrient absorption and ectomycorrhizal root formation, ultimately colonizing within fruiting bodies to form a unique bacterial microbiota. In this study, nontargeted metabolomics and whole-genome sequencing were employed to investigate the colonization characteristics of the hyphae-associated bacterium *Bacillus altitudinis* B4 on the mycelial surface of ectomycorrhizal fungus *Suillus clintonianus*, as well as the synergistic promotion of thiamine synthesis and absorption by *B. altitudinis* B4 and the fungal mycelium, respectively. The results suggested that *S. clintonianus* first secreted ureidosuccinic acid and pregnenolone, recruiting the hyphae-associated bacterium *B. altitudinis* B4 to the mycelial surface. Subsequently, the ureidosuccinic acid secreted by *S. clintonianus* further stimulated *B. altitudinis* B4 to enhance thiamine production by increasing its biomass and upregulating the expression of related functional genes. Finally, *S. clintonianus* absorbed the thiamine secreted by the *B. altitudinis* B4, promoting fungal growth and increasing the colonization rate in association with *Pinus massoniana*. This study elucidates the thiamine acquisition mechanisms of ectomycorrhizal fungi, highlighting the critical role of bacterial partners in fungal nutrition and host-fungal interactions.

## Introduction

Ectomycorrhizal (EcM) fungi establish symbiotic associations with ~6000–7000 plant species and play vital roles in forest ecosystems [1]. The EcM association enhances host plant nutrient and water uptake, improves resistance to pathogens and pollutants, and promotes seedling growth and survival [2–4]. Simultaneously, EcM fungi depend on host-derived carbon for growth, and some species can form fruiting bodies under favorable conditions [5, 6]. Thiamine, an essential cofactor in central carbon metabolism of all organisms, is partially or entirely absent in most EcM fungi, and thus needs to be obtained from external environment [7, 8]. However, some EcM fungi are thiamine-deficient and cannot effectively synthesize thiamine or acquire it from host plants, which is an critical factor limiting their growth [8, 9]. Consequently, the sources and absorption strategies of thiamine by EcM fungi in natural forest ecosystems remain a key research focus.

Previous studies have found that EcM fungi can obtain certain nutrients not only from host plants, but also from various bacterial groups with specific functions. These bacteria are selectively recruited to the surface of fungal hyphae through the secretion of specific metabolites, including organic acids, peptides, amino acids, sugars, and sugar alcohols [10–13]. The recruited bacteria may adhere and form biofilms on the hyphal surface and eventually colonize EcM fruiting bodies, forming a unique bacterial microbiota [14]. Furthermore, in complex ecosystems, certain costly metabolites, including degrading enzymes, siderophores, amino acids, and vitamins, are always considered public goods. They are produced by specific members of the community and provide benefits to all [15]. It has been reported that many soil bacteria can secrete high concentrations of thiamine [16, 17]. Typically, thiamine is synthesized from two separate parts, pyrimidine and thiazole, which are later combined in bacteria. The pyrimidine portion is converted by hydroxymethyl pyrimidine synthase (thiC) from its purine intermediate 5-aminoimidazole ribonucleotide to hydroxymethyl pyrimidine phosphate (HMP-P), which is then phosphorylated to hydroxymethyl pyrimidine pyrophosphate (HMP-PP) by hydroxymethyl pyrimidine kinase (thiD). Thiazoles are partially synthesized from glycolytic products, a sulfur carrier protein (ThiS), and glycine in a multi-step process, forming thiazole phosphate carboxylate tautomer via thiazole synthase (thiG). The compound 4-amino-5-aminomethyl-2-methylpyrimidine, produced by base degradation of thiamine, can be catalyzed by thiaminase II (tenA) as a substrate for thiD and re-enter the thiamine synthesis pathway [18, 19]. In contrast to bacterial systems, the thiamine biosynthesis pathway in plants and fungi involves a metabolically costly mechanism whereby thiazole synthase (THI4), a suicide enzyme, must sacrifice a catalytic cysteine residue at its active site to provide the sulfur atom required for thiazole ring formation [20–22]. Thus, certain hyphae-associated bacterial microbiota within EcM fruiting bodies may be closely related to the thiamine acquisition by EcM fungi.

*Suillus clintonianus* is a typical EcM fungus that can establish an EcM symbiosis with *Pinus massoniana* and produce fruiting bodies [14]. In this study, we explore the promoting effect and mechanism of *B. altitudinis* B4, isolated from the fruiting bodies, on thiamine uptake by *S. clintonianus*. Moreover, pot experiments were conducted to assess the beneficial effect of hyphae-associated bacteria on EcM formation in the host plant by promoting thiamine absorption and growth of EcM fungi.

## Materials and methods

### Microorganisms, strains, and culture conditions

The fruiting body of the EcM fungus *S. clintonianus* was collected from Thousand Island Lake in July 2019 (29°34′45.67″N, 118°54′45.71″E) [23]. The EcM fungus *S. clintonianus* and the hyphae-associated bacterium *B. altitudinis* B4 were isolated from the fruiting body. Internal tissue fragments were aseptically excised using sterile forceps to avoid contact with the outer surface, and placed onto plates containing either potato dextrose agar medium (PDA, 200 g L^−1^ potato extract, 20 g L^−1^ glucose, and 15 g L^−1^ agar, 50 mg L^−1^ streptomycin, pH 7.0) at 28°C or LB medium (tryptone 10 g L^−1^, yeast extract 5 g L^−1^, and NaCl 10 g L^−1^, pH 7.0) at 37°C, respectively. Based on the genetic distance (ITS rRNA genes) to *S. clintonianus*, additional EcM fungi (*Suillus grevillei*, 97% similarity; *Xerocomus chrysenteron*, 91%) and non-EcM fungi (*Ceriporia lacerate*, 89% similarity; *Phomopsis liquidambaris*, 81% similarity) were also used in this study. All fungi were cultivated on modified Pachlewski medium (P20, 1 g L^−1^ tartrate, 1 g L^−1^ KH_2_PO_4_, 0.5 g L^−1^ MgSO_4_, 10 g L^−1^ glucose, 1 ml L^−1^ diluted Kanieltra micronutrient solution (1:10), and 15 g L^−1^ agar, pH 5.5) at 28°C for 14 days as the fungal inoculum [13]. To obtain mycelial exudates, fungal plugs were inoculated in 50 ml of P20 medium (without thiamine) at 28°C for 14 d. The plugs were then washed twice with sterile water and transferred to 25 ml of sterile water. After 24 h, the mycelium was removed, and mycelial exudates were collected and freeze-dried after removed the mycelium by a 0.22-μm membrane (Merck Millipore, Germany) [24]. The mycelial exudates were then weighed as the original concentration and diluted to different concentrations with sterile water for the subsequent experiments.

The bacteria *Enterobacter* sp*.* and *Pseudomonas oryzihabitans*, isolated from soil, were also used. Bacteria were grown and maintained in 50 ml LB medium at 37°C for 12 h. The concentration of the bacterial inoculum (OD_600_) was adjusted using sterilized 0.9% NaCl solution after centrifugation and three washes with sterile water.

Thiamine auxotrophy assay and whole-genome sequencing of *S. clintonianus.*

The growth of EcM fungi was tested on different defined P20 media [1 g L^−1^ tartrate, 1 g L^−1^ KH_2_PO_4_, 0.5 g L^−1^ MgSO_4_, 10 g L^−1^ glucose, vitamin mix (2 μM thiamine, 2 μM riboflavin, 2 μM pantothenic acid, 2 μM pyridoxine, and 2 μM biotin), and 15 g L^−1^ agar, pH 5.5] as follows: Vitamin-, P20 without vitamin mix; VB1-, P20 without thiamine; VB2-, P20 without riboflavin; VB5-, P20 without pantothenic acid; VB6-, P20 without pyridoxine; VB7-, P20 without biotin. Fungal plugs (8 mm) were placed in the center of the plate (60 mm) and incubated at 28°C in the dark. Image J was used for measuring the mycelial area after 14 days. Mycelial density was calculated as dry weight per area.

Genomic DNA was extracted from *S. clintonianus* using a Wizard Genomic DNA Purification Kit (Promega, WI, USA). The concentration of the purified DNA was quantified with a TBS-380 fluorometer (Turner BioSystem INC., Sunnyvale, CA) and a NanoDrop 2500 spectrophotometer (Thermo Fisher Scientific, USA). Whole-genome sequencing was conducted on the HiSeq System (Illumina) and PacBio platform using a shotgun library with an insert size of 400 bp. Raw data underwent quality control processing with Fastp v0.20.0, and genome assembly was performed using Unicycler. Gene annotation was carried out with Prodigal v2.6.3. Functional annotation of the predicted coding genes was conducted against several databases, including NR, Swiss-Prot, Pfam, EggNOG, GO, and KEGG databases. The rRNA gene sequences were identified using Barrnap 0.4.2.

### Combined system of hyphae-associated bacterium and ectomycorrhizal fungus

*B. altitudinis* B4 (50 μL, OD_600_ = 0.4) was incubated into 50 ml of standard P20 medium and P20 medium supplemented with ^13^C_6_-lableled glucose (without thiamine) at 37°C, 200 rpm. After 3 days, the supernatant of *B. altitudinis* B4 was harvested by centrifugation at 8000 × g for 10 min and filtered through a 0.22-μm membrane (Merck Millipore, Germany). The contents of thiamine in the supernatant was detected by high performance liquid chromatography (HPLC, Waterse-2695, Acclaim 120 C18 column, mobile phase A: 0.1% trifluoroacetic acid, mobile phase B: acetonitrile, column temperature: 35 ± 2°C, detection wavelength: 280 nm). The flow rate was 1 ml min^−1^ with the following gradient program: 100% of mobile phase A in the first 12 min, 50% of mobile phase A, and 50% of mobile phase B in the 12–20 min. The injection volume was 5 μL. The thiamine concentration in the supernatant was also detected by the modified method [16]. The supernatant (250 μL) was filtered through a 0.22-μm membrane, and then 10 μL of 1% K_3_[Fe(CN)_6_], 150 μL of 15% sodium hydroxide solution, and 150 μL of isobutanol were added sequentially. The tubes were shaken vigorously for 1 min and centrifuged at 13 000 × g for 2 min. The upper isobutanol layer was centrifuged at 13 000 × g for 1 min after being transferred to a new 1.5 ml tube, and the fluorescence was measured under 365 nm excitation and 450 nm emission. A standard curve was plotted with a series of standard thiamine solutions. Moreover, the influence of *B. altitudinis* B4 supernatant on *S. clintonianus* was evaluated on a fresh P20 agar medium supplemented with bacterial supernatant (1:50, v/v) at 28°C. The density of mycelium was calculated after 14 days. The *S. clintonianus* (8 mm) was also inoculated into *B. altitudinis* B4 supernatant at 28°C for 7 days. The fungal mycelium was harvested, washed three times with sterile water, ground under liquid nitrogen, and lysed by ultrasonication for 30 min and centrifuged at 5000 × g for 10 min. Then, the supernatant was infused at a flow rate of 3 μL min^−1^ into a Fourier Transform Ion Cyclotron Resonance Mass Spectrometer (FTICR-MS, Solari X ESI ETD, Bruker, USA) operated in positive-ion mode with a spray voltage of 3500 V to detect ^13^C_12_-labeled thiamine (m/z 277.1).

To analyze the interaction between *S. clintonianus* and *B. altitudinis* B4 in the combined system, *S. clintonianus* (8 mm) and *B. altitudinis* B4 (50 μL, OD_600_ = 0.4) were co-cultured in P20 liquid medium (without thiamine). Treatments inoculated with *S. clintonianus* alone and *B. altitudinis* B4 alone were served as the *S. clintonianus-*only treatment and *B. altitudinis* B4-only treatment, respectively. For RT-qPCR, *S. clintonianus* was picked out and rinsed twice with sterilized ddH_2_O, from the combined treatment and *S. clintonianus*-only treatment after 7 days. *B. altitudinis* B4 was also collected by centrifuging the P20 medium from the combined treatment and *B. altitudinis* B4-only treatment. Total RNA was extracted from tissues using TRIzol reagent according to the instructions (Invitrogen, USA), and genomic DNA was removed using DNase I (TaKara, Japan). RNA quality was then determined using a 2100 Bioanalyser (Agilent, USA) and quantified using ND-2000 (NanoDrop Technologies). Only high-quality RNA samples (OD_260/280_ = 1.8–2.2, OD_260/230_ ≥ 2.0, RIN ≥ 8.0, 28S:18S ≥ 1.0, ≥ 1 μg) were selected to obtain cDNA by reverse transcription according to the kit. cDNA was obtained by reverse transcription using the AceQ qPCR SYBR Green Master Mix (Vazyme Biotech Co., Ltd, China) on a 7500 Real-Time PCR system (Applied Biosystems), and the PCR conditions were 95°C for 5 min, 95°C for 10 s, 60°C for 30 s, and 72°C for 20 s, with a total of 40 cycles. The selected genes and the primers are listed in Supplementary Table S1, and the lysis curves were checked at the end of each run to determine the specificity of the reaction. The internal reference gene was selected as *B. altitudinis* B4 16S rRNA gene and β-actin, and the expression of target genes was finally normalized by calculating the relative expression by the 2^-ΔΔCt^ method [25].

### Recruiting characteristics of ectomycorrhizal fungus to hyphae-associated bacterium

The chemotaxis and adhesion of the *B. altitudinis* B4 towards *S. clintonianus* were measured. Chemotaxis assays were performed using a soft-agar plate (0.4% agar) and a capillary method [26]. For the soft-agar assay, *B. altitudinis* B4 (4 μL, OD_600_ = 0.4) was placed on a paper disc at the center of the plate. Sterilized ddH_2_O and mycelial exudates (0.5 and 1.0 mg ml ^−1^) were added to both sides apart from bacteria (1 cm), respectively. All plates were incubated at 30°C for 48 h, and the chemotactic response was evaluated based on the orientation of bacterial growth. For the capillary assay, *B. altitudinis* B4 inoculum (250 μL, OD_600_ = 0.4) was added to individual well of a 96-well microtiter plate. Capillary tubes, sealed at one end, were filled with 10 μL of different concentrations of hyphal exudates and immersed in the bacterial suspension. Sterilized ddH_2_O served as the control. The colony-forming units (CFUs) of *B. altitudinis* B4 inside the capillaries were counted after incubation for 60 min at 28°C.

The swimming and swarming motility of *B. altitudinis* B4 was also assessed in the study [27]. *B. altitudinis* B4 (1 μL, OD_600_ = 0.4) was inoculated at the center of swimming medium (1% tryptone, 0.5% NaCl, 0.25% glucose, 0.3% agar) or swarming medium (1% tryptone, 0.5% yeast extract, 0.5% NaCl, 0.5% glucose, 0.5% agar) containing 0.1, 0.5, and 1.0 mg ml ^−1^ of mycelial exudates, respectively. Colony diameters were determined after 8 h (swimming) and 20 h (swarming) of incubation at 30°C, respectively. Sterilized ddH_2_O was used as the control.

The adhesion of *B. altitudinis* B4 to *S. clintonianus* mycelia was also examined. *S. clintonianus* was first cultured on solid P20 medium (without thiamine) for 1 week at 28°C. Then, *B. altitudinis* B4 (1 μL, OD_600_ = 0.4) was inoculated at 1 cm from the edge of *S. clintonianus* colony, and a glass slide was inserted diagonally at a distance of 1 cm apart from the bacteria. After 28 days, the slide was observed with a Zeiss Axio Imager A1 microscope (Zeiss, Jena, Germany) and a scanning electron microscope (JEOL, Japan). Two other bacteria, *Enterobacter* sp. and *P. oryzihabitans*, were served as controls. Hyphal colonization was assessed via microscope to compare bacterial adhesion patterns. In addition, glass fibers (with or without mycelial exudates of *S. clintonianus*) were used instead fungal hyphae to test whether mycelial exudates could recruit bacteria, causing them to move along the hyphae. Detailed experimental protocols had been provided in the Supplementary Materials.

### Non-targeted metabolomic analysis of *S. clintonianus* exudates

Specific compounds in *S. clintonianus* exudates related to the chemotaxis, colonization, and thiamine secretion of *B. altitudinis* were investigated using nontargeted metabolomics. Exudates of the EcM fungus *S. grevillea* and the non-EcM fungus *C. lacerate* were also included as controls. Fungal exudates (1 ml) were freeze-dried and re-suspended in pre-chilled 80% methanol by well vortex. Then, the samples were incubated on ice for 5 min and centrifuged at 15 000 × g, 4°C for 15 min. The supernatant was diluted to a final concentration of 53% methanol. The samples were used for LC–MS/MS analysis after being centrifuged at 15 000 × g, 4°C for 15 min.

Non-targeted metabolomic analysis was performed using a Vanquish UHPLC system (Thermo Fisher, Germany) coupled with an Orbitrap Q Exactive TM HF mass spectrometer/Orbitrap Q Exactive TMHF-X mass spectrometer (Thermo Fisher, Germany) in Novogene Co., Ltd (Beijing, China). Samples were injected onto a Hypersil Gold column (100 × 2.1 mm, 1.9 μm) using a 12-min linear gradient at a flow rate of 0.2 ml min^−1^. The eluents for the positive and negative polarity modes were eluent A (0.1% FA in water) and eluent B (methanol). The solvent gradient was set as follows: 2% B, 1.5 min; 2%–85% B, 3 min; 85%–100% B, 10 min; 100%–2% B, 10.1 min; 2% B, 12 min. Orbitrap Q Exactive TM HF mass spectrometer was operated in positive/negative polarity mode with a spray voltage of 3.5 kV, the capillary temperature of 320°C, sheath gas flow rate of 35 psi, aux gas flow rate of 10 L min^−1^, S-lens RF level of 60, and aux gas heater temperature of 350°C.

Raw data files generated by UHPLC–MS/MS were processed using the Compound Discoverer 3.3 (CD3.3, Thermo Fisher, Germany) to perform peak alignment, peak picking, quantitation, metabolite identification, and relative quantification. The metabolomics data were submitted to the EMBL-EBI MetaboLights database (MTBLS11889).

### Pot experiment design

The pot microcosm composed of two main compartments: a plant compartment and a hyphal compartment, separated by a 45 μm nylon mesh. Soil and vermiculite were mixed in a 3:1 ratio in both compartments after sterilized by autoclaving at 121°C for 2 h (Fig. S1). Soil was collected from the top 20 cm of a forest plot at Nanjing Normal University (Nanjing, China, 32°11′N, 118°91′E). Its physicochemical properties were shown in Table S2.

*P. massoniana* seeds were surface-sterilized with 70% (v/v) ethanol and 3% (v/v) sodium hypochlorite for 3 min, followed by three washes with sterile water. The surface-sterilized seeds were germinated on sterilized vermiculite at 28°C in the dark. Seedlings were transplanted into the plant compartment after 30 days. Moreover, *S. clintonianus* inoculum (5 g) and *B. altitudinis* B4 inoculum (1 ml, OD_600_ = 0.4) were added to the hyphal compartment when needed in different treatments. There were four different treatments: control treatment (CK, autoclaved *S. clintonianus* and autoclaved *B. altitudinis* B4); *S. clintonianus*-only treatment (Sc, inoculation with fungus and autoclaved bacteria); *B. altitudinis* B4-only treatment (B4, inoculation with bacteria and autoclaved fungus); and combined treatment (Sc + B4, inoculation with *S. clintonianus* and *B. altitudinis*). All treatments were conducted in a controlled environment greenhouse (28°C, 16/8 h light/dark, with light at 450 μmol m^−2^ s^−1^) at Nanjing Normal University (Nanjing, China, 32°11′N, 118°91′E) for 90 days.

The growth index of the *P. massoniana* seedlings was measured at 90 days post-inoculation. Chlorophyll was extracted from fresh leaves and measured according to the spectrophotometric method [28]. Root activity of *P. massoniana* seedlings wss determined using commercial assay kits according to the manufacturer’s instructions (Edison Biotechnology Co., Ltd, China). Phosphorus (P) content was determined following established protocols [29–31]. Ectomycorrhizal structure was observed with a Zeiss Axio Imager A1 microscope (Zeiss, Germany) after staining with Propidium Iodide (Sigma-Aldrich) and Wheat Germ Agglutinin (Thermo Fisher, Germany) [32].

The biomass and ectomycorrhizal colonization rate of *S. clintonianus* were measured [33]. For quantitative RT-PCR, the methods are described in the Supplementary Materials, and the genes and primers are listed in Table S1. All quantitative PCR amplifications were conducted on an Applied Biosystems 7500 Real-Time PCR system using AceQ Universal SYBR qPCR Master Mix (Vazyme, China).

### Statistical analysis

Statistical analyses were performed using GraphPad Prism 8 software (version 8.0). Comparisons between two groups were performed using an unpaired two-tailed Student’s *t-*test. Differences among multiple groups were compared using one-way analysis of variance, as only one independent variable was involved in each comparison. Tukey’s post hoc tests were performed to determine significant differences. Data were expressed as the mean with standard error (SE). Results with a value of *P* < .05 were considered to indicate statistical significance.

## Results

### *S. clintonianus* is auxotrophic for thiamine

**Figure 1:**
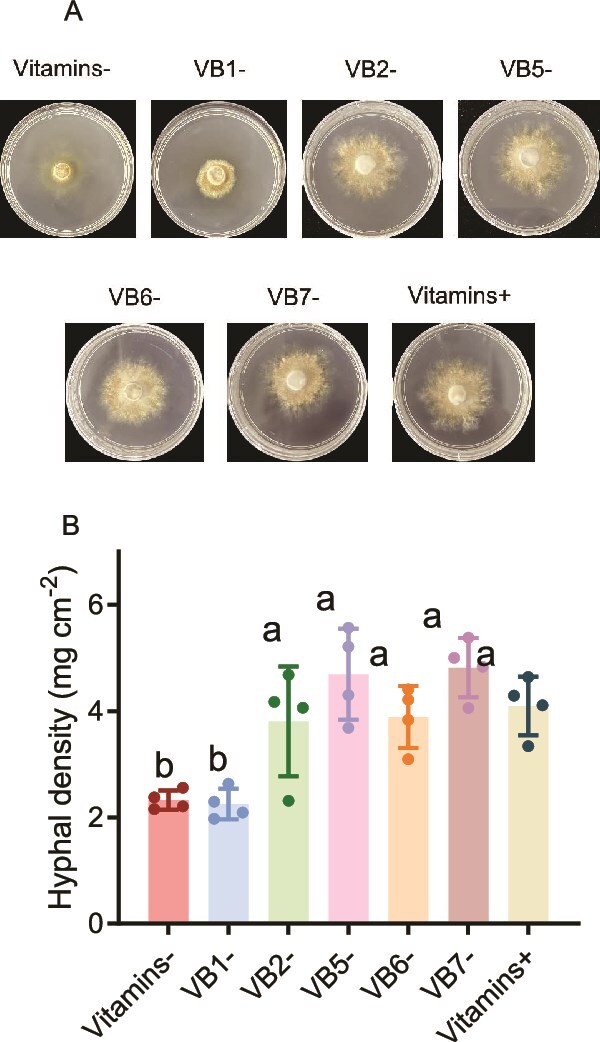
The growth of *S. clintonianus* under different conditions. (A) Growth of *S. clintonianus* on agar plates in the defined medium without a particular vitamin, as shown in each row. (B) Hyphal density of fungal grown on each agar plate. + indicates supplementation of the corresponding vitamin in the culture; − indicates no supplementation. Data and error bars are the mean ± SE (n = 4), and different letters indicate significant differences among the different treatments (one-way analysis of variance with Tukey’s test, *P* < .05).

When thiamine, riboflavin, pantothenic acid, pyridoxine, and biotin were individually removed from the culture medium, the mycelial density of *S. clintonianus* was most inhibited in the absence of thiamine, showing a 45.2% reduction in mycelial density compared to the complete vitamin supplement (Fig. 1A and B). This confirmed that exogenous thiamine promotes the mycelial growth of *S. clintonianus.* However, the thiamine precursor hydroxymethylpyrimidine (HMP) was added to the culture medium, no recovery in the growth of *S. clintonianus* was observed. Furthermore, *S. grevillea* also exhibited similar thiamine auxotrophy compared to *S. clintonianus*, whereas *X. chrysenteron* did not (Fig. S2).

To investigate whether *S. clintonianus* can synthesize thiamine de novo, we sequenced its genome with a combination of Illumina short read and PacBio long read technologies. Functional annotation was conducted to identify genes associated with thiamine biosynthesis. The assembled genome of *S. clintonianus* was 6.18 Gb in size with a GC content of 49.30% and contained 18 412 open reading frames (ORFs). Genomic analysis showed that *S. clintonianus* lacks key thiamine biosynthesis genes, indicating it cannot synthesize thiamine de novo (PRJNA 1355154, GenBank, NCBI). However, we identified a putative thiamine transporter (*THI73*) that may be involved in the uptake of exogenous thiamine.

### Hyphae-associated bacterium *B. altitudinis* B4 secretes thiamine to promote the growth of *S. clintonianus*

**Figure 2:**
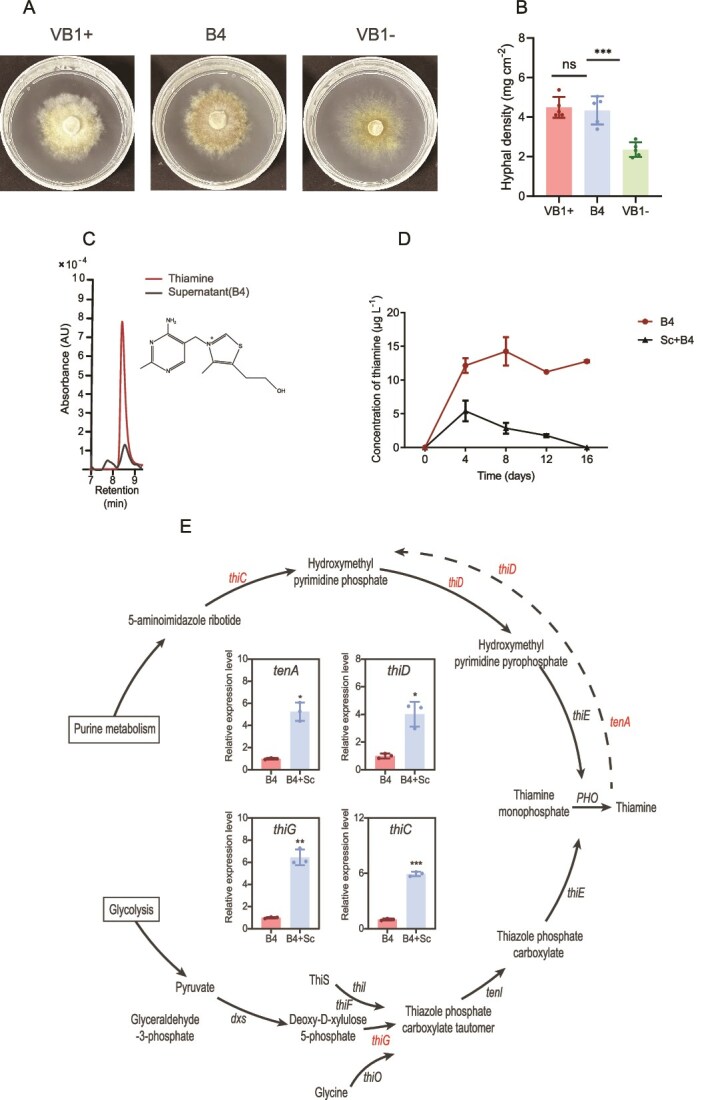
*B. altitudinis* B4 secretes thiamine to promote the growth of *S. clintonianus*. (A) Growth of *S. clintonianus* on agar plates in the defined medium with different additives (VB1+: with thiamine; B4: the supernatant of *B. altitudinis* B4; VB1-: without thiamine). (B) Hyphal density of *S. clintonianus* grown on each agar plate. (C) HPLC image of the supernatant of *B. altitudinis* B4. (D) The concentration of thiamine in the culture media (B4: *B. altitudinis* B4 cultured alone; Sc + B4: *B. altitudinis* B4 cultured with *S. lintonianus*). (E) Thiamine metabolic pathway and relative expression of genes related to thiamine metabolism in *B. altitudinis* B4. Red genes indicate significant difference between the B4 treatment and Sc + B4 treatment. Black genes indicate no significant difference. Data and error bars are the mean ± SE (n = 3), and asterisks indicate significant differences between the two groups (unpaired two-tailed unpaired Student’s *t*-test, ^*^P < .05, ^**^P < .01, ^***^P < .001).

The supernatant of *B. altitudinis* B4 significantly alleviated thiamine deficiency and promoted the hyphal growth of *S. clintonianus* (Fig. 2A and B), whereas its volatile organic compounds had no significant effect (Fig. S3). Thiamine was detected in the culture medium under the *B. altitudinis* B4-only treatment and the combined treatment (Fig. 2C). The concentration of thiamine in the culture medium increased rapidly within 4 days in both treatments, reaching 13.2 μg L^−1^ in the *B. altitudinis* B4-only treatment and 4.43 μg L^−1^ in the combined treatment (Fig. 2D). Subsequently, the thiamine concentration stabilized in the *B. altitudinis* B4-only treatment but gradually decreased in the combined treatment. This phenomenon might be due to *S. clintonianus* absorbing and utilizing the thiamine secreted by *B. altitudinis* B4. Additionally, ^13^C_12_-thiamine was detected within the *S. clintonianus* hyphae following their cultivation with metabolites from *B. altitudinis* B4 that had been cultured in ^13^C_6_-glucose-supplemented medium (Fig. S4A). RT-qPCR analysis also revealed that the expression of thiamine metabolism-related genes *thiD, thiG, tenA*, and *thiC* in *B. altitudinis* B4 was up-regulated by 4.01, 6.44, 5.25, and 5.94-fold, respectively, in the combined treatment compared to the *B. altitudinis* B4-only treatment (Fig. 2E). Conversely, the expression of *THI73* in *S. clintonianus* was upregulated by 1.86-fold in the combined treatment compared to the *S. clintonianus*-only treatment (Fig. S4B).

### *S. clintonianus* recruits *B. altitudinis* B4 to hyphosphere

**Figure 3:**
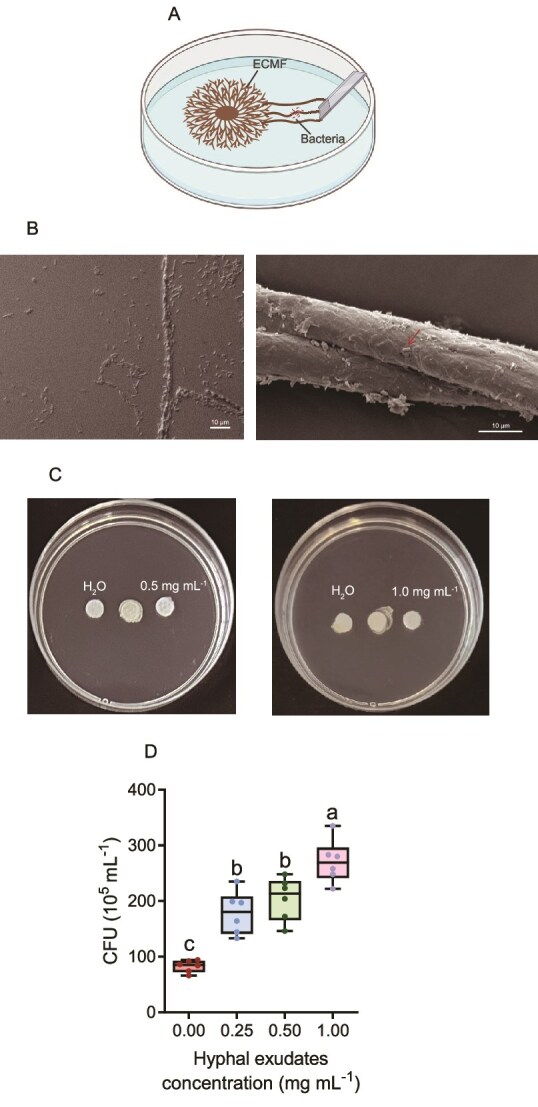
Effects of *S. clintonianus* exudates on *B. altitudinis* B4 chemotaxis and movement. (A) Schematic diagram of the experimental setup (EcMF: ectomycorrhizal fungi). (B) *B. altitudinis* B4 on the hyphae surface of *S. clintonianus* was observed by light microscopy and scanning electron microscopy (The red arrow points to *B. altitudinis* B4). (C) Chemotactic response of *B. altitudinis* B4 toward different concentrations of *S. clintonianus* exudates. Sterilized ddH_2_O was served as the control. (D) Chemotactic response of *B. altitudinis* B4 toward different concentrations of *S. clintonianus* exudates by the capillary method. Data and error bars are the mean ± SE (n = 6), and different letters indicate significant differences among the different treatments (one-way analysis of variance with Tukey’s test, *P* < .05).

To test the chemotactic ability of *B. altitudinis* B4 toward *S. clintonianus, S. clintonianus* was inoculated in the center of an agar plate, *B. altitudinis* B4 was inserted 1 cm apart from the fungal plug, and a glass slide was inserted diagonally at a distance of 1 cm from the bacteria (Fig. 3A). The results showed that *B. altitudinis* B4 could adhere to *S. clintonianus* hyphae and simulate mycelial networks (glass fiber with mycelial exudates of *S. clintonianus*) (Fig. 3B, S5A–C). No *B. altitudinis* B4 was observed on glass fiber with sterilized ddH_2_O (Fig. S5B). The result suggested that *B. altitudinis* B4 exhibited robust attachment to mycelial exudates, with dense bacterial aggregates clearly visible on hyphal surfaces (Fig. S5A). In contrast, *Enterobacter* sp. and *P. oryzihabitans* showed negligible hyphal colonization under identical conditions (Fig. S5D, 5E). Moreover, the mycelial secretions of *S. clintonianus* were collected from medium with different carbon concentrations to study their effect on *B. altitudinis* B4. The results showed that *B. altitudinis* B4 moved towards the side of mycelial secretions even collected from low carbon environment (Fig. S6A), with the chemotactic response strengthening as the exudate concentration increased (Fig. 3C). Capillary chemotaxis assays also confirmed these results, showing a 32.7% greater attraction at 1.0 mg ml^−1^ mycelial exudates concentration compared to 0.5 mg ml^−1^ (Fig. 3D). The swimming and swarming motility of *B. altitudinis* B4 were significantly enhanced on the media containing *S. clintonianus* mycelial exudates compared to the control (Fig. S6B, 6C). This enhanced motility was accompanied by a 2.19-fold and 1.66-fold upregulation of key chemotaxis genes *filG* and *MCP*, respectively (Fig. S6D). Thus, those results suggested that the mycelial exudates of *S. clintonianus* specifically attract and enhance the motility of *B. altitudinis* B4. Furthermore, chemotaxis experiment indicated that the mycelial exudates of EcM fungus *S. grevillei* also had a chemotactic effect on *B. altitudinis* B4, whereas the EcM fungus *X. chrysenteron* and the non-EcM fungi (*C. lacerata* and *P. liquidambaris*) did not (Fig. S7). Thus, this suggested that specific EcM fungi might secrete particular compound to recruit specialized bacteria under the thiamine-deficient conditions.

**Figure 4:**
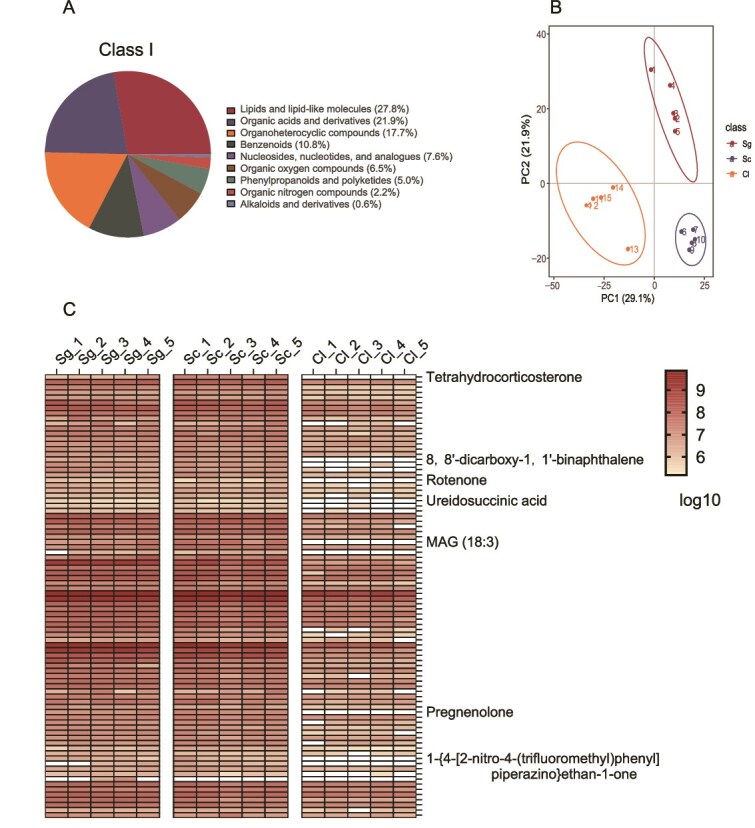
Comparison of the mycelial exudates of *S. clintonianus, S. grevillei*, and *C. lacerata*. (A) The components of mycelial exudates. (B) Principal component analysis (PCA) of the mycelial exudates of *S. clintonianus* (Sc), *S. grevillei* (Sg), and *C. lacerata* (Cl). (C) Heatmap showing the relative amounts of 86 substances with up-regulated levels of ectomycorrhizal fungi in each treatment. The red color indicates high relative levels, and the white color indicates that the substance was not detected. Seven substances detected only in mycelial exudates of ectomycorrhizal fungi are shown on the right side of each column. Each treatment contains 5 replicates.

To identify these compounds, nontargeted metabolomics was performed on exudates from *S. clintonianus* (Sc), *S. grevillei* (Sg), and *C. lacerata* (Cl) under thiamine deficiency. The main mycelial exudates from all three fungi were lipids and lipid-like molecules (Fig. 4A). The PCA plots showed significant metabolite profile differences between treatments (Fig. 4B). There were 469 differentially abundant metabolites between Sc and Cl treatment (Fig. S8A) and 428 between Sg and Cl treatment (Fig. S8B). In addition, 219 metabolites were common differential metabolites, of which 86 metabolites were upregulated in Sc and Sg treatment (Fig. S8C). Heatmap analysis of the relative contents of 86 metabolites revealed 7 compounds specific to the two EcM fungi, including tetrahydrocorticosterone, ureidosuccinic acid, pregnenolone, monoacylglycerides, 1-{4-[2-nitro-4-(trifluoromethyl)phenyl] piperazino}ethan-1-one, 8,8′-dicarboxy-1,1′-binaphthalene, and rotenone (Fig. 4C). Thus, these seven metabolites were correlated with bacterial chemotaxis, motility, and thiamine production (Fig. S8D).

**Figure 5:**
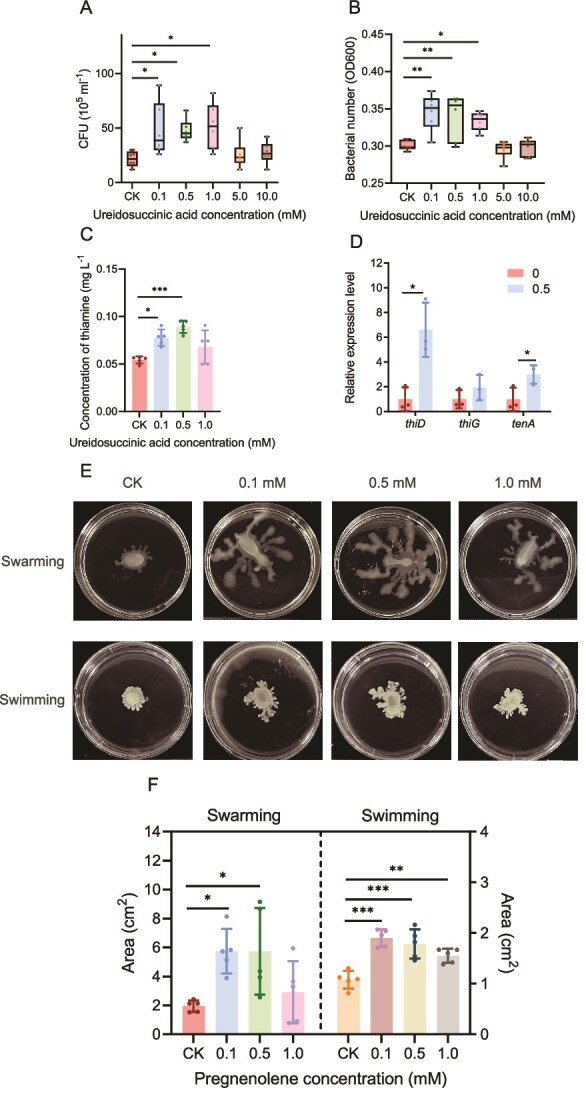
Effects of ureidosuccinic acid and pregnenolone on *B. altitudinis* B4. (A) Effect of different concentrations of ureidosuccinic acid on the bacterial growth of *B. altitudinis* B4 (CK: no ureidosuccinic acid in the medium). (B) Chemotactic response of *B. altitudinis* B4 toward different concentrations of ureidosuccinic acid by the capillary method. Sterile water was served as the control (CK). (C) The concentration of thiamine in the supernatant treated with different concentrations of ureidosuccinic acid (CK: no ureidosuccinic acid in the medium). (D) Relative expression of genes related to thiamine metabolism in *B. altitudinis* B4 when treated with different concentrations of ureidosuccinic acid (0: no ureidosuccinic acid in the medium; 0.5: with 0.5 mM ureidosuccinic acid in the medium. (E and F) Effects of different concentrations of pregnenolone on *B. altitudinis* B4 motility (CK: no pregnenolone in the medium). Data and error bars are the mean ± SE (n = 5), and asterisks indicate significant differences between the two groups (unpaired two-tailed Student’s *t*-test, ^*^P < .05, ^**^P < .01, ^***^P < .001).

*In vitro* experiments confirmed that ureidosuccinic acid and pregnenolone directly promoted the chemotaxis and motility of *B. altitudinis* B4 (Fig. 5). Capillary assays confirmed the chemotactic effects of ureidosuccinic acid on *B. altitudinis* B4 (Fig. 5A). The results showed that ureidosuccinic acid also promoted the growth of *B. altitudinis* B4 at 0.1, 0.5, and 1.0 mM (Fig. 5B). Moreover, the concentration of thiamine in the supernatant of *B. altitudinis* B4 was observed to increase in response to the presence of ureidosuccinic acid at concentrations of 0.1 and 0.5 mM (Fig. 5C). The expression levels of *thiD* and *tenA*, essential for thiamine synthesis in *B. altitudinis* B4, were also significantly elevated in the presence of 0.5 mM ureidosuccinic acid (Fig. 5D). However, pregnenolone could only improve the bacterial motility, including swimming and swarming of *B. altitudinis* B4 (Fig. 5E and F). These findings indicated that different metabolites in the mycelial exudates of *S. clintonianus* might play distinct roles in the combined system.

### B. *altitudinis* B4 promotes the establishment of the ectomycorrhizal symbiosis between *S. clintonianus* and *P. massoniana.*

**Figure 6:**
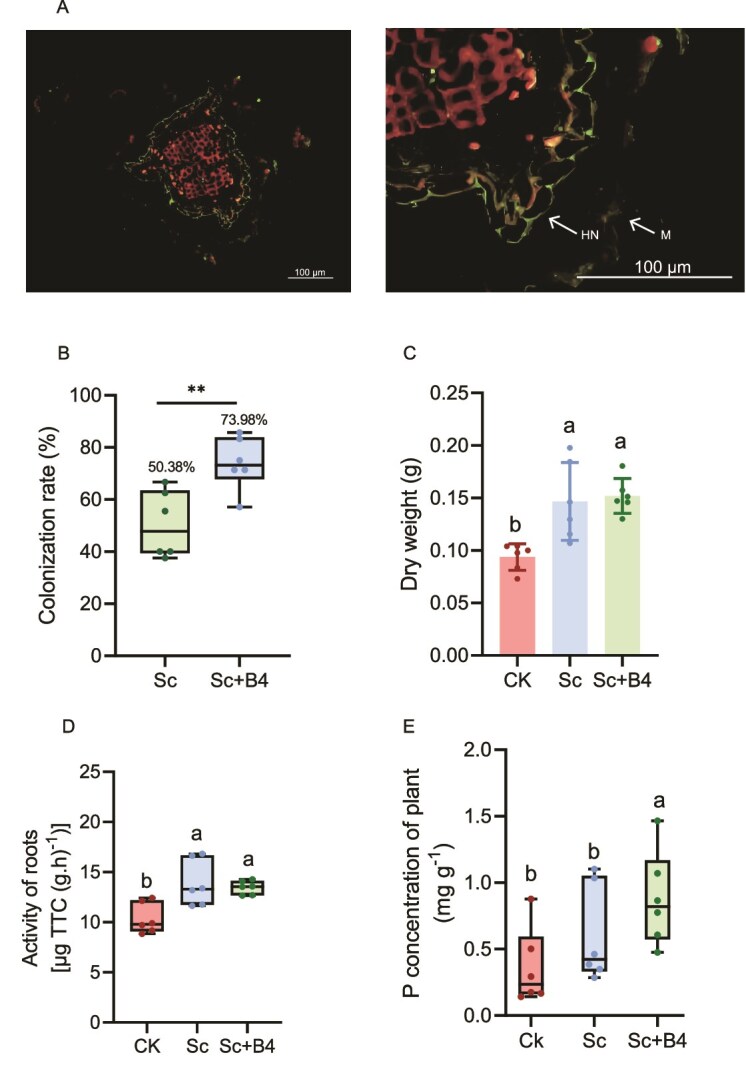
*B. altitudinis* B4 promotes the establishment of an ectomycorrhizal system between *S. clintonianus* and *P. massoniana*. (A) Representative images of a transverse cross-sections of *P. massoniana* seedling lateral roots colonized by *S. clintonianus* (M, mantle; HN, Hartig net, cortical cells stained red, mycelium stained green). (B) Mycorrhizal rate of the *S. clintonianus*-only treatment (Sc) and the combined treatment (Sc + B4). Asterisks indicate significant differences between the two groups (unpaired two-tailed unpaired Student’s *t*-test, ^**^P < .01). (C–E) Dry weight, root activity, and phosphorus content of seedlings. CK, inoculation with autoclaved *S. clintonianus* and autoclaved *B. altitudinis* B4; Sc, inoculation with fungus and autoclaved *B. altitudinis*; Sc + B4, inoculation with *S. clintonianus* and *B. altitudinis*. Data and error bars are the mean ± SE (n = 6), and different letters indicate significant differences among the different treatments (one-way analysis of variance with Tukey’s test, *P* < .05).

A partitioned pot experiment was conducted to investigate the effect of *B. altitudinis* B4 on ectomycorrhizal formation (Fig. S1). After 90 days, it was found that under both *S. clintonianus*-only and combined treatments, *P. massoniana* seedlings could form ectomycorrhizal structures with *S. clintonianus*, including dichotomously branched roots, mantles, and Hartig nets (Fig. S9A; Fig. 6A). The relative biomass of *S. clintonianus* in both the hyphal and plant compartments was higher in the combined treatment than in the *S. clintonianus*-only treatment (Fig. S9B). The ectomycorrhizal colonization rate was also significantly higher in the combined treatment (Fig. 6B). Moreover, the dry weight, root activity, and phosphorus content of *P. massoniana* were significantly greater in the combined treatment compared to the *S. clintonianus*-only treatment (Fig. 6C–E). In addition, *B. altitudinis* B4 alone had no significant effect of on seedlings growth (e.g. biomass, height) or key physiological indicators (e.g. chlorophyll content, nutrient uptake) compared to the control (Fig. S10). Thus, these results indicated that *B. altitudinis* B4 promoted the mycelia growth of *S. clintonianus*, which was beneficial for the formation of the ectomycorrhizal symbiosis between *S. clintonianus* and *P. massoniana* seedlings.

## Discussion

In natural soil environments, nutrient-deficient microorganisms can obtain essential nutrients by forming cross-kingdom partnerships to access metabolites produced by other microbes [10, 34]. In this study, we found that *S. clintonianus* lacks the key genes for thiamine synthesis but retains genes encoding thiamine transporters and activation enzymes. This is consistent with the general pattern of thiamine metabolism across kingdoms: most prokaryotes (e.g. *Bacillus* spp.) possess complete thiamine biosynthesis pathways, whereas fungi exhibit high variability. Yeasts are able to synthesize thiamine *de novo*, whereas other fungi, such as *S. clintonianus*, are auxotrophic [9, 19, 35, 36]. Under thiamine-deficient conditions, the EcM fungus *S. clintonianus* auxotrophy for thiamine restored mycelia growth when cocultured with the hyphae-associated bacterium *B. altitudinis* B4 or its metabolites. Previous studies have shown that diverse organisms, from fungi to plants, often acquire thiamine through cross-kingdom exchange rather than de novo synthesis, which may be attributed to the loss of thiamine biosynthesis genes. The loss of thiamine biosynthesis genes may be related to long-term evolution adaptation to the high energy consumption stress for thiamine production [37, 38]. For example, *Aspergillus nidulans* can use the thiamine synthesized by *B. subtilis* to save energy [39]. The endophytic fungus *Serendipita indica*, which is auxotrophic for thiamine, obtains thiamine from the metabolites of *B. subtilis* to support its growth [16]. Similarly, rice (*Oryza sativa* L.) can also assimilate the thiamine secreted by the endophytic *Streptomyces hygroscopicus* OsiSh-2, thereby conserving energy required and enhancing resistance to *Fusarium oxysporum* [40].

Little is known about the compounds that are involved in the construction of combined system between EcM fungi and hyphae-associated bacteria in terms of communication [41, 42]. In natural soil, the chemotaxis of hyphae-associated bacteria toward EcM fungi may be the first step for adhesion and colonization. In this study, hyphae-associated bacterium *B. altitudinis* B4 showed significant chemotaxis to the exudates of specific EcM fungus rather than non-EcM fungi. Compare with *C. lacerata*, the exudates of *S. clintonianus* had higher levels of several readily utilizable compounds that were always related to bacterial chemotaxis, such as methionine and tryptamine (Fig. S11A). However, chemotaxis assays revealed that methionine and tryptamine had no chemotaxis towards *B. altitudinis* B4 (Fig. S11B). Subsequent nontargeted metabolomic analysis identified seven compounds unique to the specific EcM fungal exudates under thiamine-deficient conditions. Among these, ureidosuccinic acid played a major role in selectively recruiting *B. altitudinis* B4, whereas pregnenolone mainly enhanced bacterial motility. Previous study showed that ureidosuccinic acid was involved in nitrogen metabolism processes, which might be related to bacterial chemotaxis [43, 44]. For instance, in *Azospirillum brasilense*, chemotaxis signaling is connected to nitrogen metabolism through the core chemotaxis proteins CheA1 and CheA4 [44], suggesting a possible mechanism for ureidosuccinic acid-mediated recruitment. Pregnenolone, a steroid, can modulate membrane fluidity as a membrane component and regulate gene transcription as a signaling molecule [45]. Fungi may synthesize pregnenolone via oxidative cleavage of cholesterol side chains, similar to the pathway in vertebrates [46]. It is reported that pregnenolone can maintain microtubule abundance and promote cell motility [47], which is partially similar to the enhancement of bacterial motility by pregnenolone in our study, but the underlying mechanism requires further investigation. Nevertheless, we cannot exclude the presence of other compounds relevant to chemotaxis in the exudates. This warrants further comparative metabolomics studies on a wider array of EcM and non-EcM fungi.

**Figure 7:**
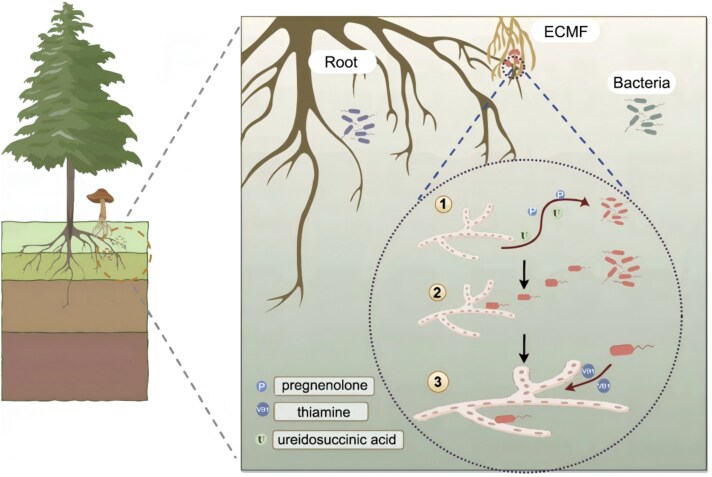
Ectomycorrhizal fungi recruit hyphae-associated bacterium to promote their growth. First, ectomycorrhizal fungi secrete ureidosuccinic acid, pregnenolone, and other substances to recruit certain bacteria. Second, the bacteria are attracted and adhere to ectomycorrhizal fungal hyphae. Third, the bacteria secret thiamine to promote the growth of hyphae.

After chemotaxis and colonization, a combined system was established between EcM fungi and hyphae-associated bacteria [14, 24]. In this study, the EcM fungus *S. clintonianus* could promote the thiamine secretion of *B. altitudinis* B4 in the combined system by increasing the biomass and the expression of thiamine metabolism-related functional genes of *B. altitudinis* B4, including *thiD, thiG, thiC*, and *tenA*. In bacteria, *thiD, thiG*, and *thiC* are key genes in the thiamine biosynthetic pathway [19], and *tenA* is involved in salvaging of base-degraded thiamine [48, 49]. In the combined system, the expression levels of these key genes were up-regulated, which implies that more thiamine was produced by *B. altitudinis* B4 to supply *S. clintonianus*. Moreover, previous studies showed that EcM fungi can secrete sugars and organic acids, etc., to support bacteria for growth [13, 50, 51]. For example, trehalose released by EcM fungus *Laccaria bicolor* S238N improves survival and induces chemotaxis in *P. fluorescens* BBc6R8 [13]. Phenylacetic acid, a common metabolite released at fungal surfaces and enriched in EcM-associated soils, can be utilized by bacteria for energy [50]. However, some symbiotic associations with vertical transmission, such as *Dictyostelium discoideum* farming symbiosis, can allow an organism to acquire new traits provided by its partner without partner-finding adaptations [52]. In our study, we did not specifically examine the presence of *B. altitudinis* B4 in fungal spores to indicate the possibility of vertical transmission. Although we did not examine whether *B. altitudinis* B4 is vertically transmitted via fungal spores, we observed that ureidosuccinic acid secreted by EcM fungus *S. clintonianus* may regulate pyrimidine and purine biosynthesis [43]. Because the pyrimidine ring is biosynthetic intermediate of thiamine, the increased availability of this precursor could further stimulate thiamine production by *B. altitudinis* B4.

Ectomycorrhizal fungi establish mutualistic symbioses with host plants through a multi-stage process. Initially, fungal hyphae aggregate on the root surface, penetrate and subsequently colonize the root tissues. Ultimately, a functional symbiosis is achieved, characterized by formation of Hartig net and bidirectional nutrient exchange between the partners [53]. In particular, a specific group of bacteria termed “Mycorrhiza Helper Bacteria” (MHB) are known to facilitate ectomycorrhizal formation [11]. In this study, the hyphae-associated bacterium *B. altitudinis* B4 could also increase the ectomycorrhizal colonization rates between *S. clintonianus* and *P. massoniana* seedlings in pot experiments. Previous studies had also found that high levels of thiamine can increase the infestation rate of rust fungus *Uromyces fabae* [54]. Thus, the *B. altitudinis* B4 might promote the mycelial growth of *S. clintonianus* by supplying thiamine, thereby enhancing ectomycorrhizal formation in our study. Furthermore, *P. massoniana* seedlings grew significantly better in both the combined treatment and *S. clintonianus*-only treatment compared to other treatments, which was also consistent with the previously reported finding that EcM fungi contribute to host growth by facilitating the uptake of soil minerals, such as N, P, etc [55–57].

Previous research indicates that certain bacteria can migrate along fungal hyphae (“hyphal highways”) to colonize plant roots [58, 59]. These bacteria may enhance plant growth both indirectly by facilitating nutrient mobilization, and directly via modulation of plant physiology through bacterial-derived metabolites such as phytohormones and antibiotics [60–62]. However, according to our study, the absence of direct effects from root-inoculated *B. altitudinis* B4 suggested that the observed enhancement in plant performance was primarily mediated by the EcM fungi through the formation of functional mycorrhizal structures, which improve nutrient acquisition (particularly phosphorus). Thus, *B. altitudinis* B4 likely functions as a mycorrhization helper bacterium, promoting the establishment of these beneficial fungal-root associations rather than directly influencing plant physiology. However, it remains unclear what benefits the bacteria gain in this consortium. Tracer experiments analyzing the movement of labeled compounds would help clarify it in the future study.

Overall, our results indicate that *S. clintonianus*, in a thiamine-deficient environment, might secrete ureidosuccinic acid and pregnenolone to recruit *B. altitudinis* B4 to the hyphosphere. This bacterium, in turn, enhanced the growth of *S. clintonianus* by supplying thiamine, thereby promoting the formation of the ectomycorrhizal symbiosis (Fig. 7). We propose that the ectomycorrhizal system may be a ternary symbiosis, wherein EcM fungi may recruit specific soil bacteria to form a distinctive bacterial microbiota within their fruiting bodies, potentially aiding in the completion of their life cycle. This study provides preliminary insights into the intricate interactions within ectomycorrhizal ecosystems, contributing to a broader understanding of fungal ecology.

## Supplementary Material

wraf290_Supplementary_Materials_R3

## Data Availability

The of whole-genome sequencing of *S. clintonianus* has been deposited under GenBank PRJNA1355154. Metabolomics data were submitted to the EMBL-EBI MetaboLights database (MTBLS11889). All data supporting the findings of this study are available in the manuscript or supplementary information.
