# Supplementary material for: Ectomycorrhizal fungi recruit hyphae-associated bacteria that metabolize thiamine to promote pine symbiosis
Source: ISME J. 2025 Dec 27;20(1):wraf290. doi: 10.1093/ismejo/wraf290 (PMC12815266; doi:10.1093/ismejo/wraf290)
Supplement: wraf290_Supplementary_Materials_R3 [file wraf290_supplementary_materials_r3.doc]

**Ectomycorrhizal fungi recruit hyphae-associated bacteria that metabolize thiamine to promote pine symbiosis**

**Running head: EcM fungi acquire thiamine from bacteria**

Jiale Zhu^1^, Mengya Yu^1^, Tingyu Zheng^1^, Jie Zhang^1^, Genyue Cao^1^, Xiaohan Wu^1^, Chuanchao Dai^1^, Yaseen Ullah^1^, Wei Zhang^1^*, Yong Jia^1^*

^1^Jiangsu Key Laboratory of Pathogenesis and Ecosystems, Jiangsu Engineering and Technology Research Center for Industrialization of Microbial Resources, College of Life Sciences, Nanjing Normal University, Nanjing, 210023, Jiangsu, China

* Corresponding authors: Wei Zhang and Yong Jia

Email: zhwnjnu@163.com and jiayong_2001@njnu.edu.cn

Address: Jiangsu Key Laboratory of Pathogenesis and Ecosystems, Jiangsu Engineering and Technology Research Center for Industrialization of Microbial Resources, College of Life Sciences, Nanjing Normal University, Nanjing, 210023, Jiangsu, China

**
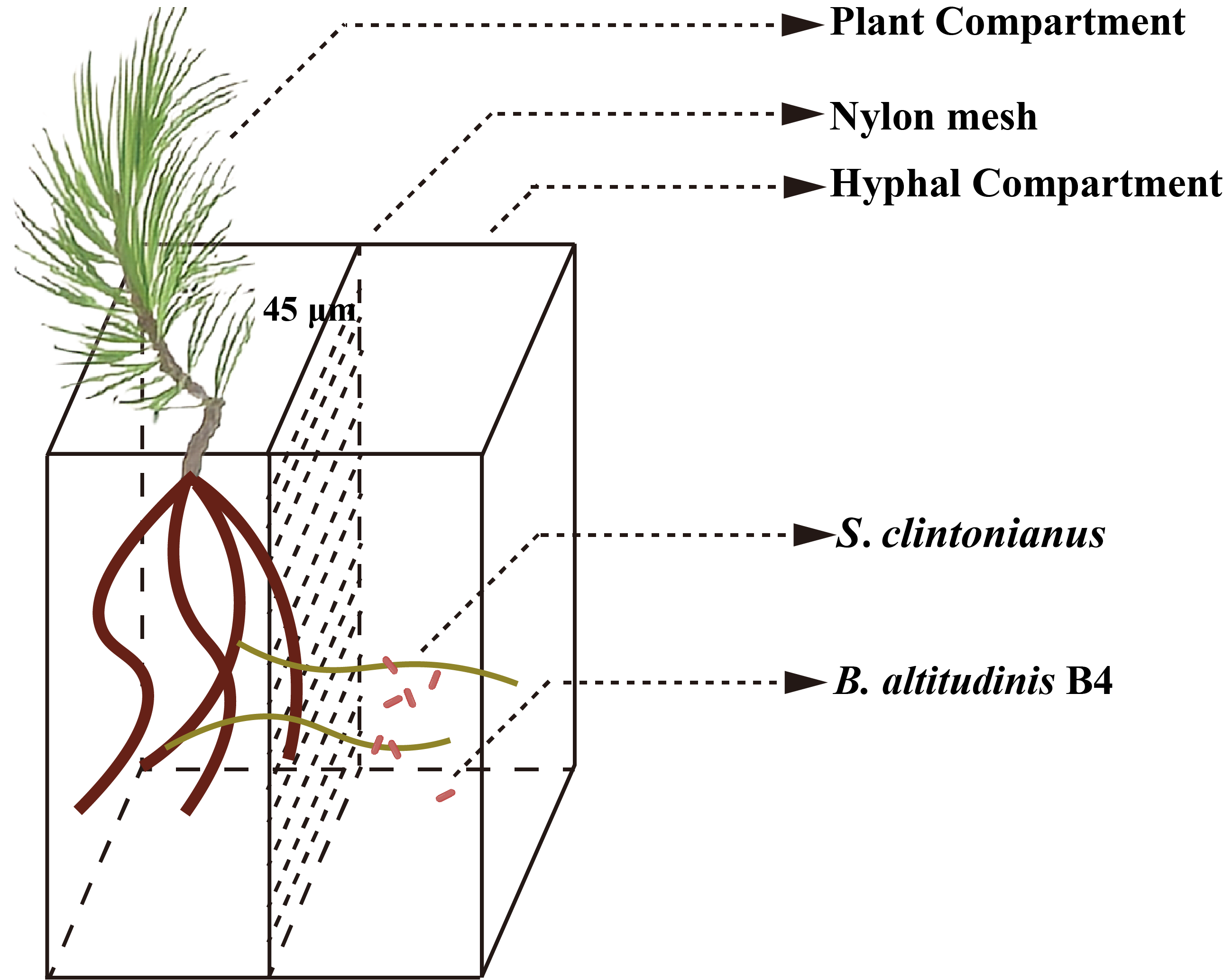
**

**Fig. S1. Schematic diagram of the potting experiment.** The microcosm of the pot was composed of two main compartments: plant compartment and hyphal compartment. Two compartments were separated by 45 μm nylon mesh. Soil and vermiculite were mixed in a 3:1 ratio in both compartments.

**
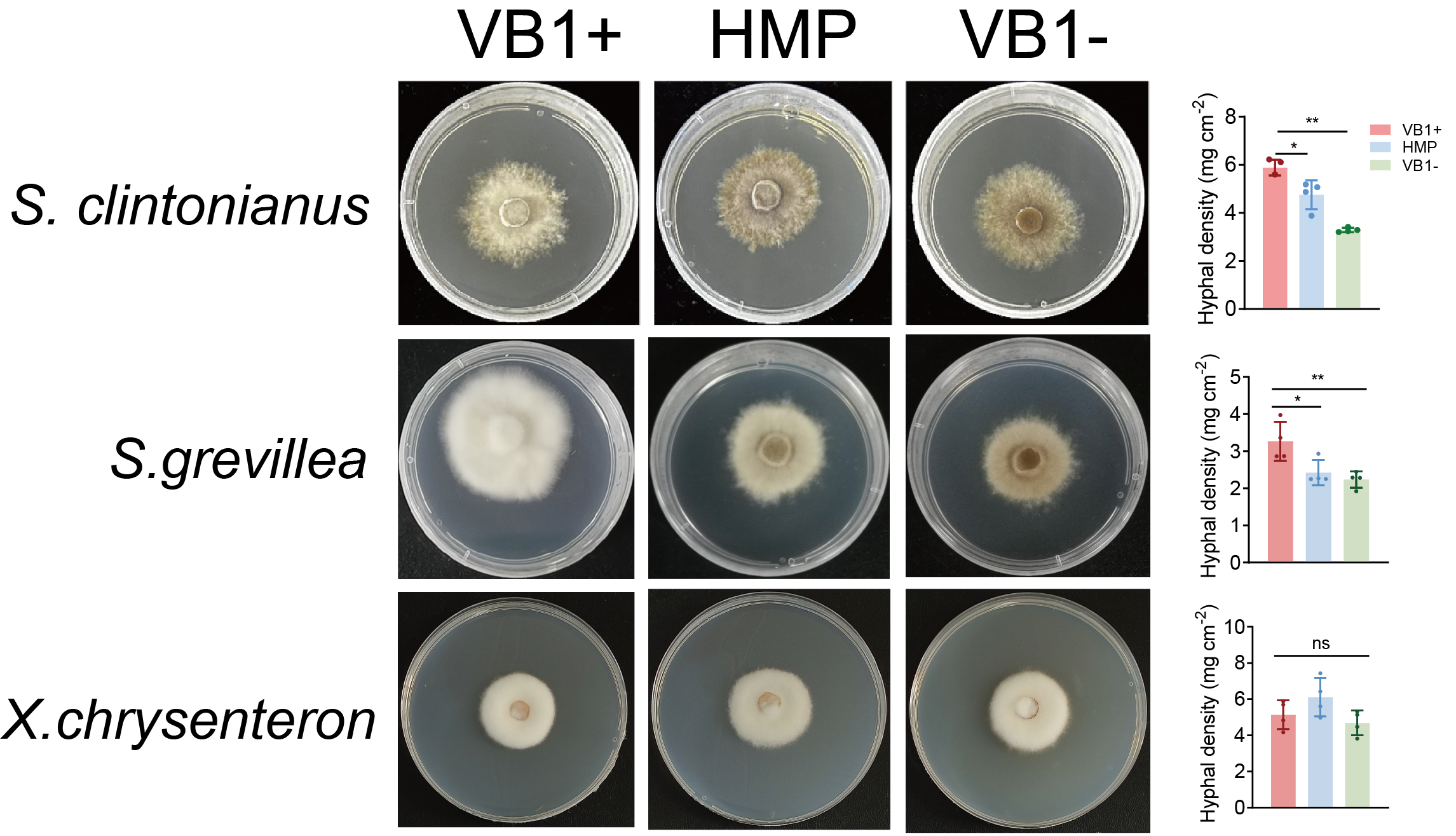
**

**Fig. S2. Thiamine requirements of different ectomycorrhizal fungi.** Growth and hyphal density of *S. clintonianus*, *S. grevillea*, and *X. chrysenteron* on agar plates in the defined medium with different additives (VB1+: with thiamine; HMP: thiamine precursors; VB1-: without thiamine). Data and error bars are the mean ± SE (n = 3) and asterisks indicate significant differences between the two groups (unpaired two-tailed Student’s *t*-test, **P* < 0.05, ***P* < 0.01).


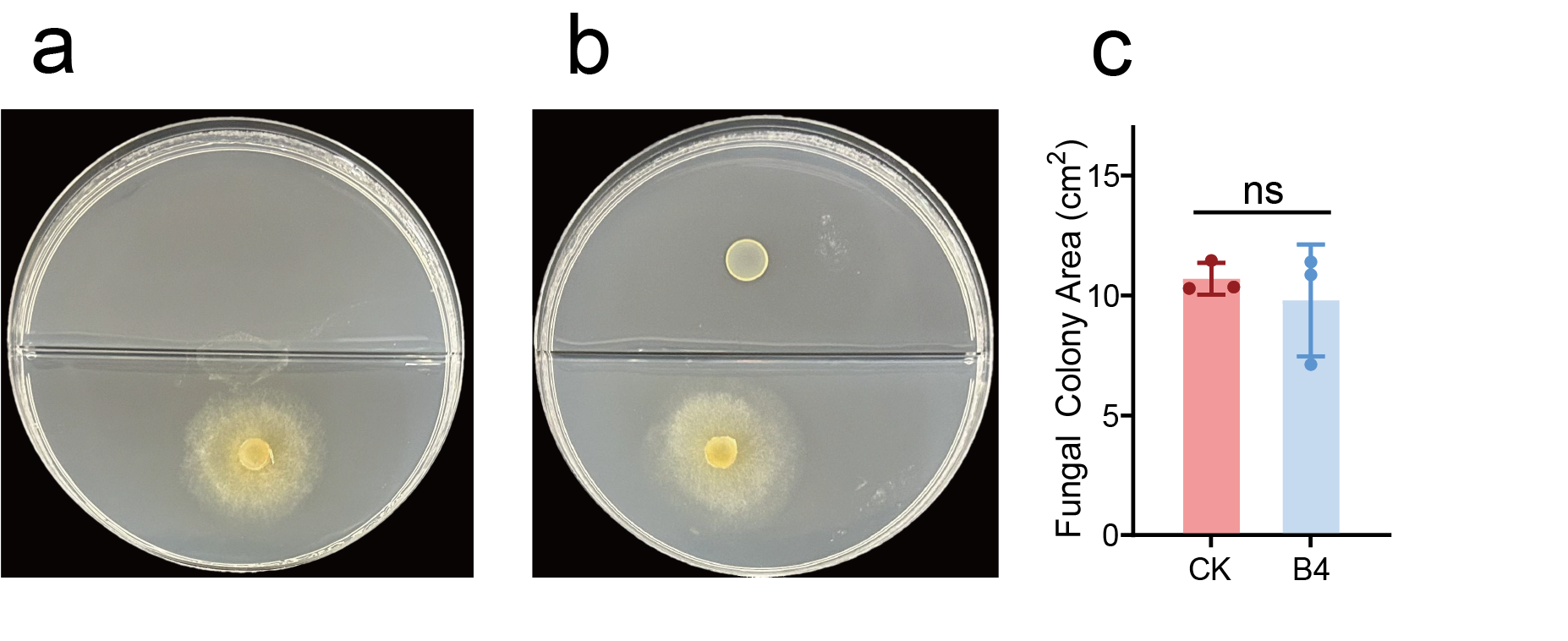


**Fig. S3. Effects of *B. altitudinis* B4 organic volatiles on *S. clintonianus*.** (a) *S. clintonianus* cultured alone. (b) *S. clintonianus* cultured with *B. altitudinis* B4. (c) Fungal colony area growth on agar plates (CK: *S. clintonianus* cultured alone; B4: *S. clintonianus* cultured with *B. altitudinis* B4). Data and error bars are the mean ± SE (n = 3), and “ns” indicate no significant differences between the two groups (Two-tailed unpaired Student’s *t*-test, *p* > 0.05).


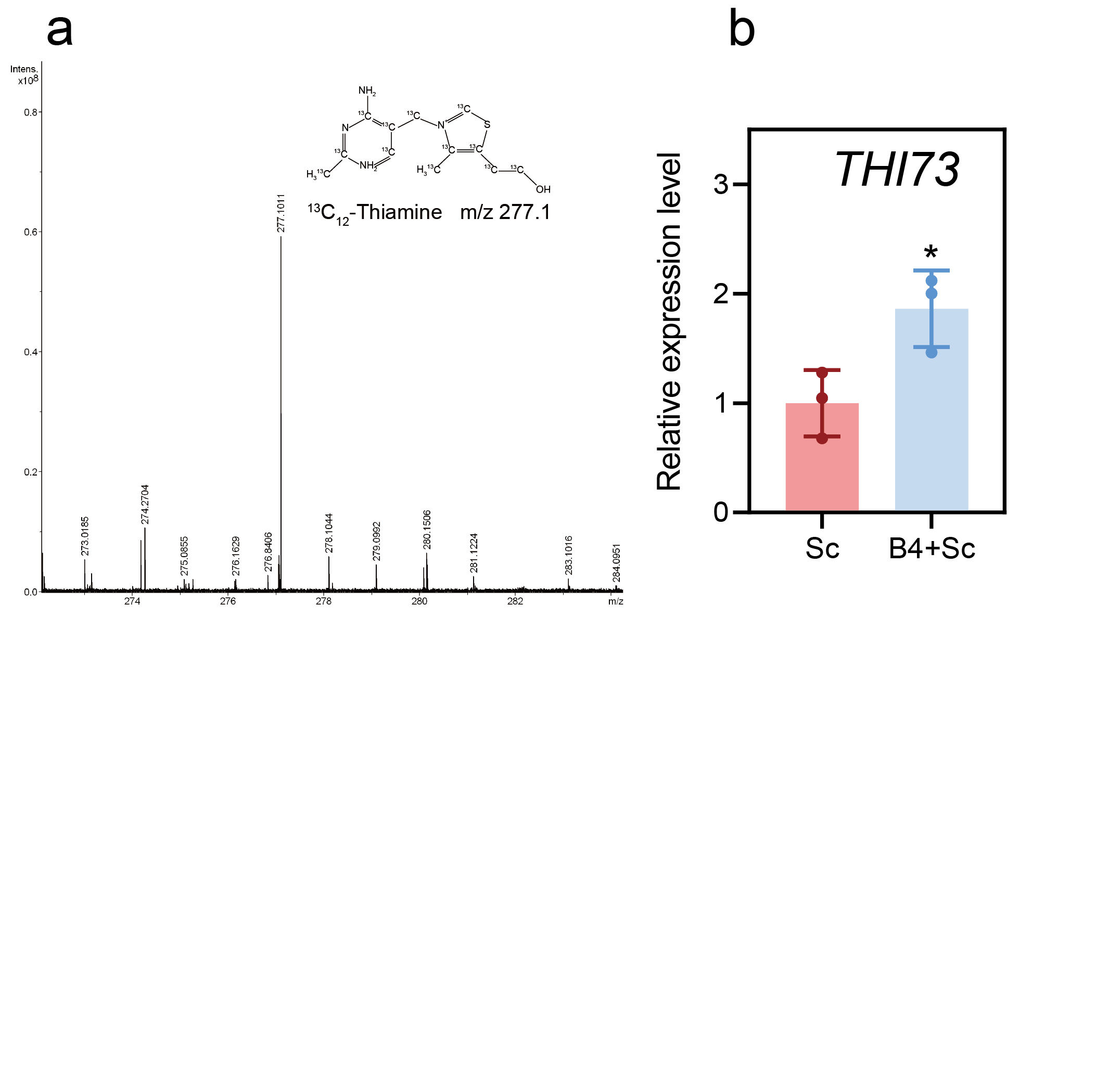


**Fig. S4. The ^13^C_12_-thiamine and relative expression levels of *THI73* in *S. clintonianus*.** (a) ^13^C_12_-thiamine in *S. clintonianus* hyphae. (b) Relative expression levels of *THI73* in *S. clintonianus*. Sc: *S. clintonianus* cultured alone; B4+Sc: *S. clintonianus* cultured with *B. altitudinis* B4. Data and error bars are the mean ± SE (n = 3), and asterisks indicate significant differences between the two groups (Two-tailed unpaired Student’s *t*-test, **P* < 0.05).


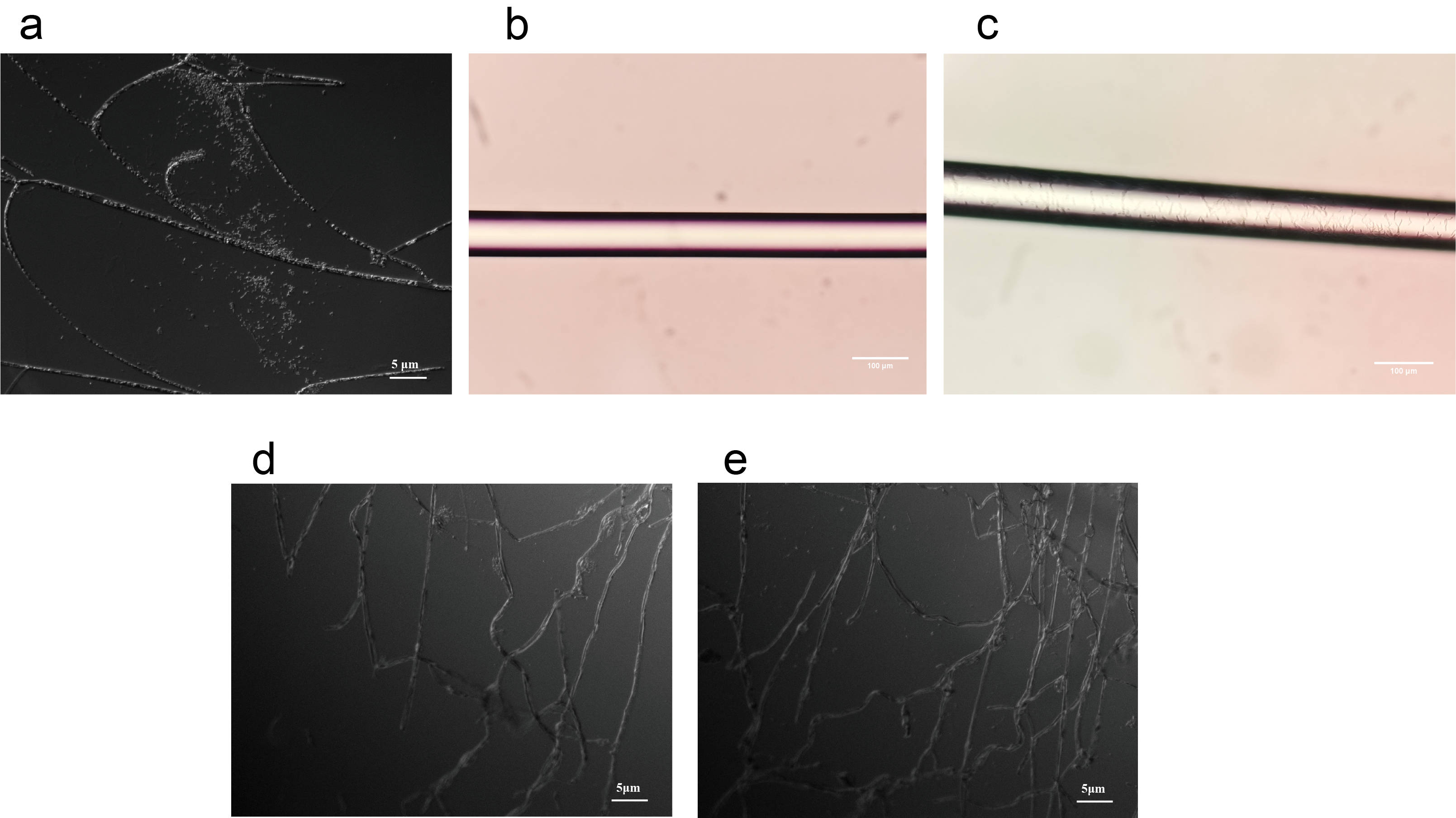


**Fig. S5. Adhesion of different bacterial strains co-cultured with *S. clintonianus* mycelium.** (a) Attachment of *B. altitudinis* B4 to *S. clintonianus* hyphae. (b) Glass fiber with sterilized ddH_2_O. (c) Glass fiber with mycelial exudates of *S. clintonianus*. (d) Attachment of *Enterobacter* sp. on *S. clintonianus* hyphae. (e) Attachment of *P. oryzihabitans* on *S. clintonianus* hyphae.


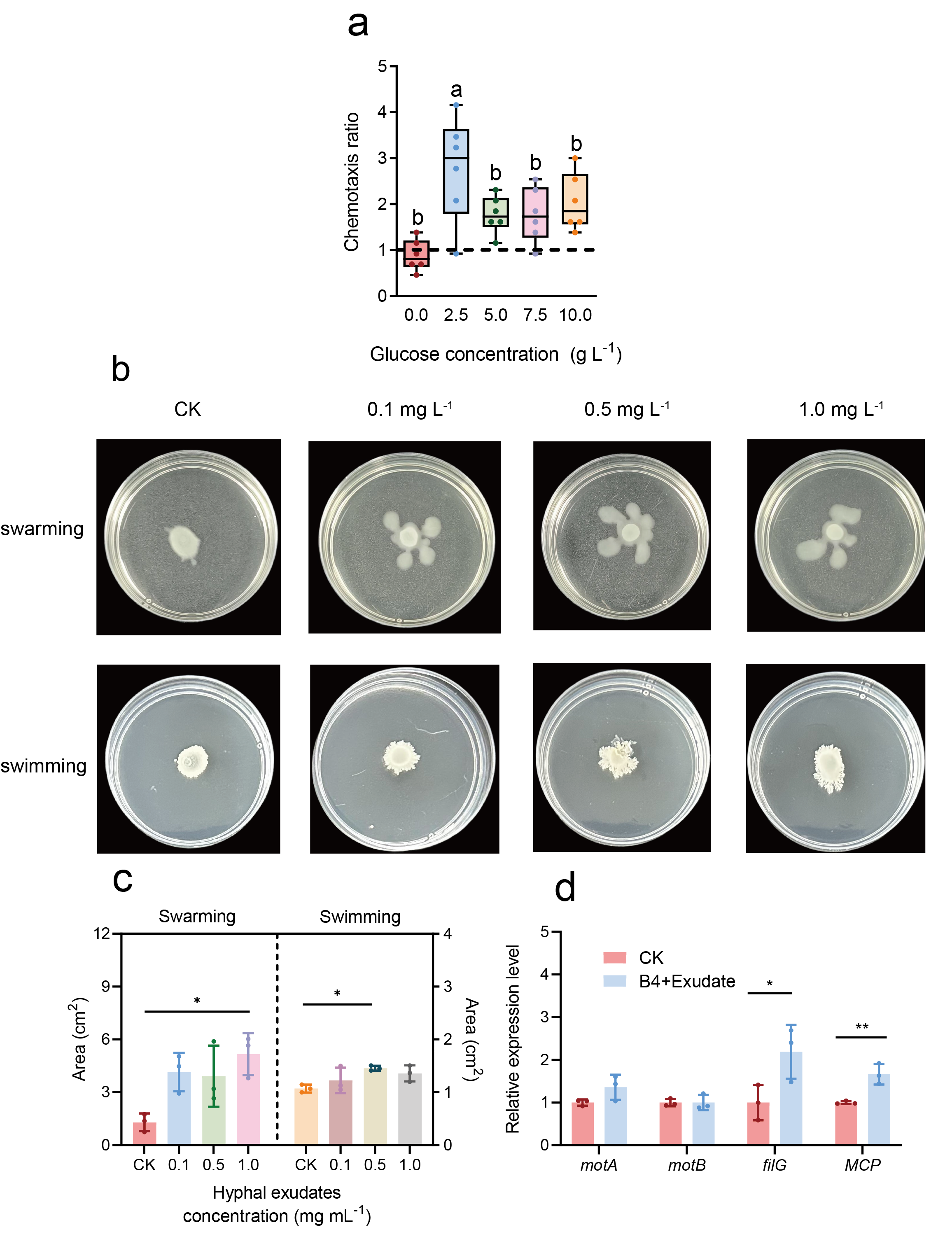


**Fig. S6. Effects of *S. clintonianus* exudates on *B. altitudinis* B4 chemotaxis.** (a) Capillary assay for the chemotactic response of *B. altitudinis* B4 to mycelial exudates of *S. clintonianus* incubated at different glucose concentrations. When the chemotaxis ratio is greater than 1, it indicated greater chemotaxis to *B. altitudinis* B4 than the sterile water control. (b, c) Effects of different concentrations of *S. clintonianus* exudates on *B. altitudinis* B4 motility (CK: no *S. clintonianus* exudates in the medium). (d) Expression levels of genes related to chemotaxis and flagellar motility in *B. altitudinis* B4 (CK: no *S. clintonianus* exudates in the medium; B4+Exudate: 0.5 mg mL^-1^ *S. clintonianus* exudates in the medium). Data and error bars are the mean ± SE (n = 6). Different letters indicate significant differences among the different treatments (one-way analysis of variance with Tukey’s test, *P* < 0.05). Asterisks indicate significant differences between the two groups. (unpaired two-tailed Student’s *t*-test, **P* < 0.05, ***P* < 0.01).

**
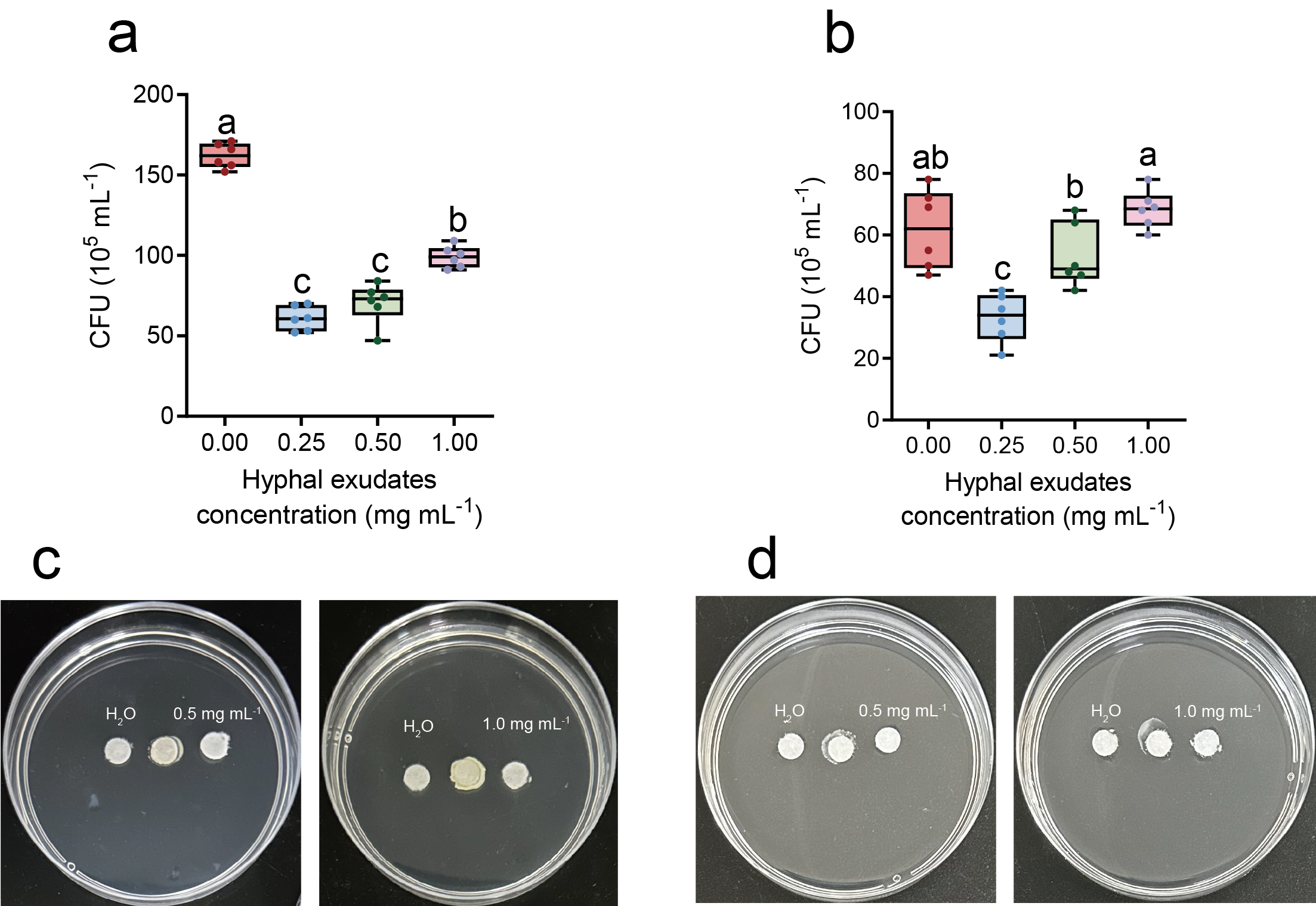
**

**Fig. S7. Effects of fungal exudates on *B. altitudinis* B4 movement.** (a) Capillary assay for the chemotactic response of *B. altitudinis* B4 to mycelial exudates of *X. chrysenteron*. (b) Capillary assay for the chemotactic response of *B. altitudinis* B4 to mycelial exudates of *P. liquidambaris*. (c) Chemotactic response of *B. altitudinis* B4 toward different concentrations of *S. grevillei* exudates. (d) Chemotactic response of *B. altitudinis* B4 toward different concentrations of *C. lacerata* exudates. Data and error bars are the mean ± SE (n = 6) and different letter indicate significant differences among the groups (one-way analysis of variance with Tukey’s test, *P* < 0.05).

**
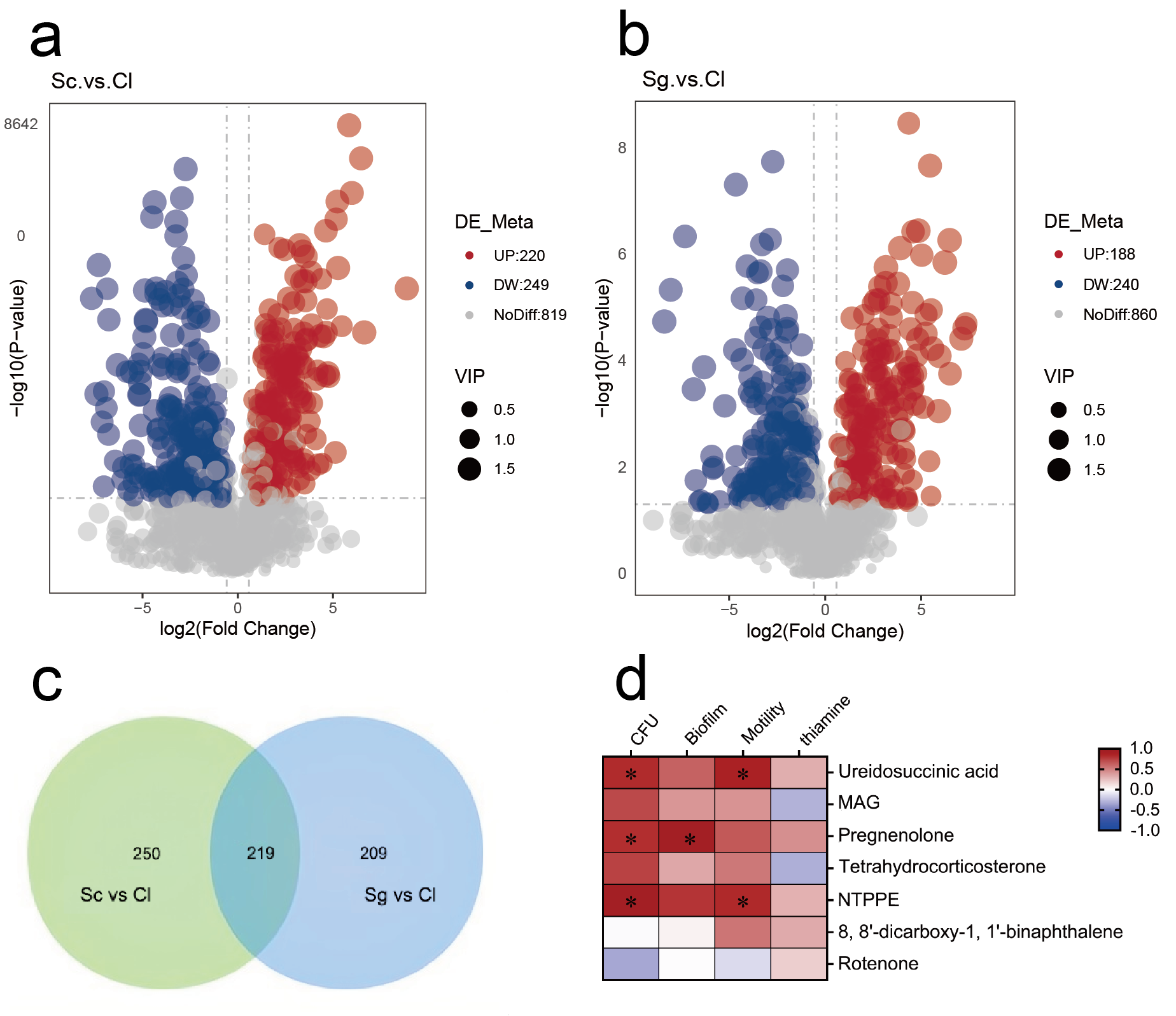
**

**Fig. S8. Comparisons of the mycelial secretions of *S. clintonianus*, *S. grevillei*, and *C. lacerata*.** (a) Volcano diagram demonstrating differential metabolites in the mycelial secretions of *S. clintonianus* (Sc) compared to those of *C. lacerata* (Cl). (b) Volcano diagram demonstrating differential metabolites in the mycelial secretions of *S. grevillei* (Sg) compared to those of *C. lacerata* (Cl). (c) Common differential metabolites between Sc vs Cl and Sg vs Cl (219 metabolites are common differential metabolites between Sc vs Cl and Sg vs Cl.) (d) Relationships between seven metabolites and bacterial chemotaxis, biofilm, motility and thiamine production.

**
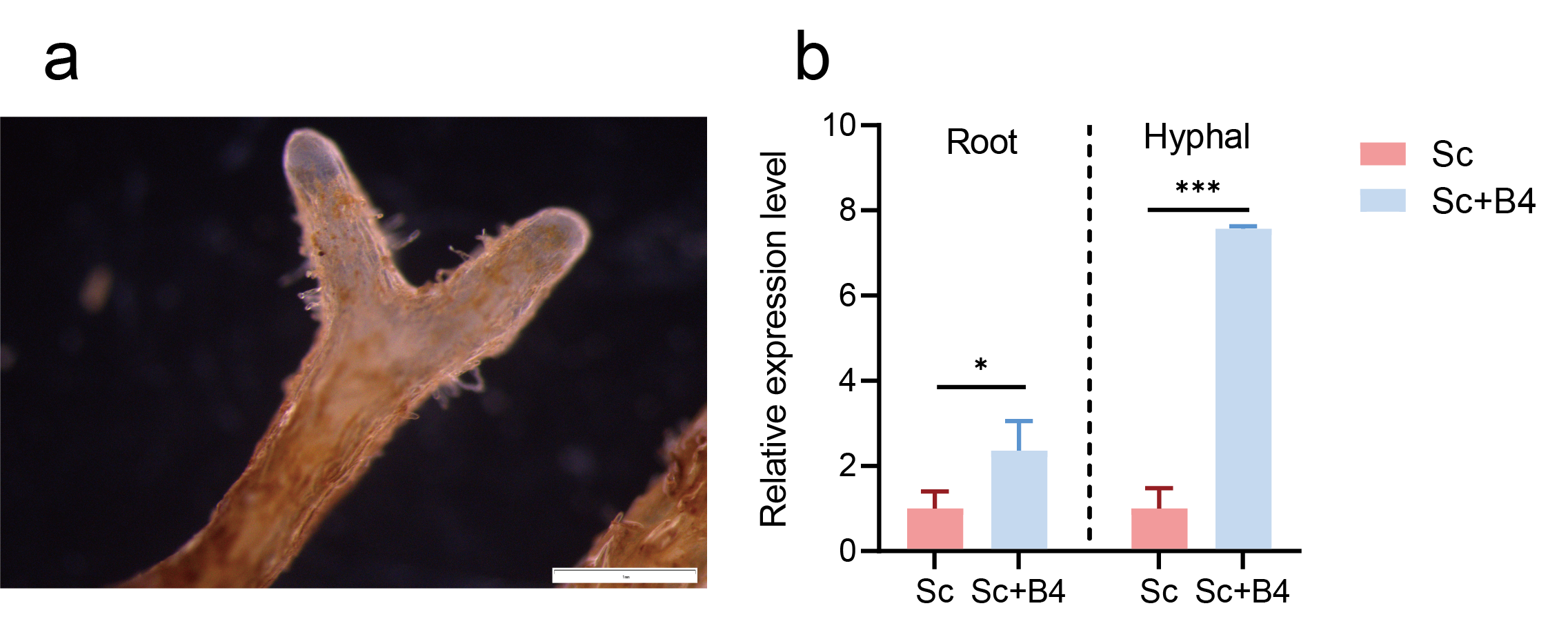
**

**Fig. S9. *B. altitudinis* B4 promotes the establishment of an ectomycorrhizal system between *S. clintonianus* and *P. massoniana*.** (a) Micrograph image of a *P. massoniana* seedlings lateral root colonized by *S. clintonianus*. (b) The relative biomass of *S. clintonianus* of the *S. clintonianus*-only treatment (Sc) and the combined treatment (Sc+B4). Data and error bars are the mean ± SE (n = 3), and asterisks indicate significant differences between the two groups. (unpaired two-tailed Student’s *t*-test, **P* < 0.05, ****P* < 0.001).


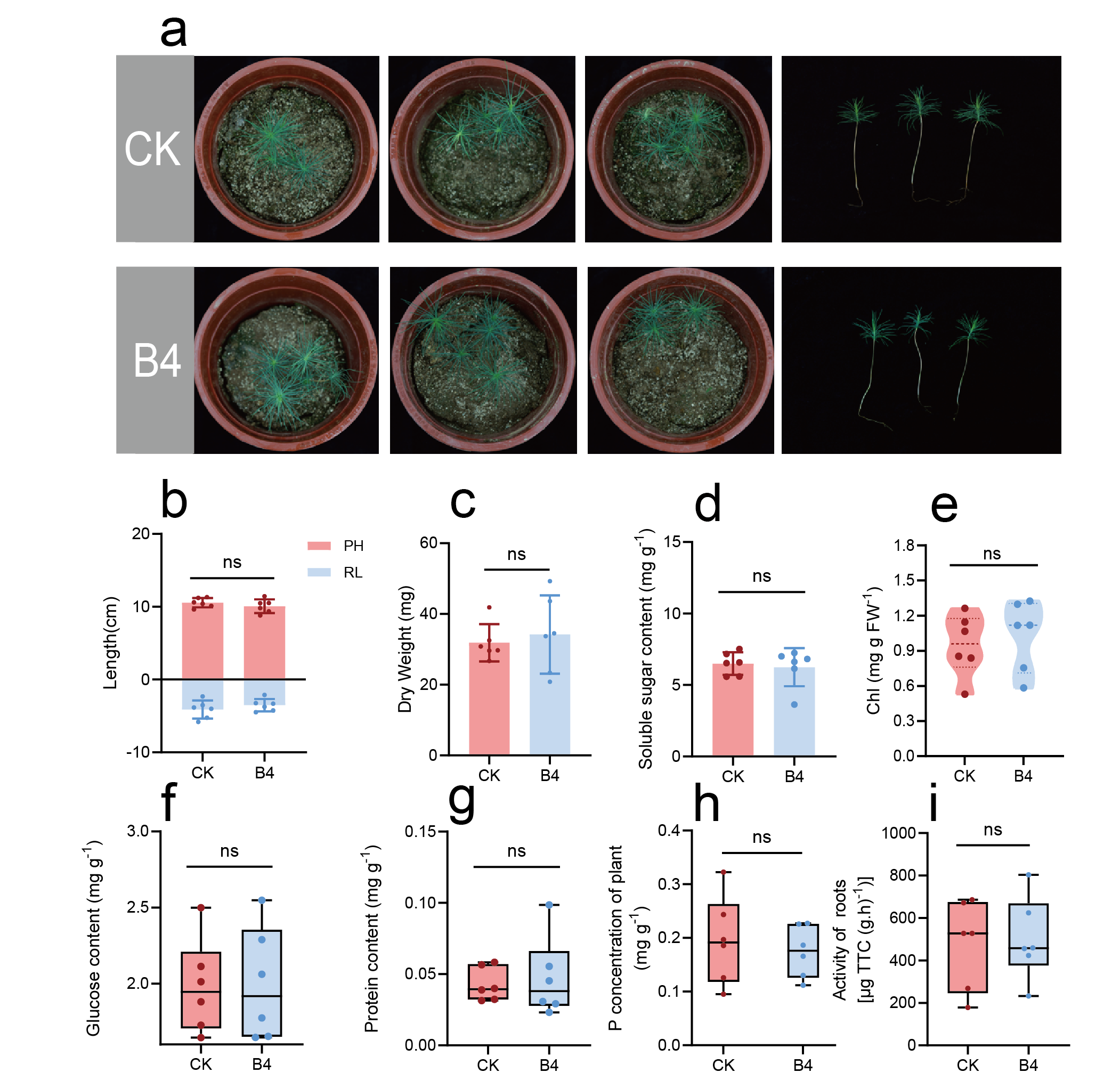


**Fig. S10. Physiological indices of *P. massoniana* seedlings inoculated with *B. altitudinis* B4 alone.** (a) Representative pictures of *P. massoniana* seedlings. (b) Plant length and root length of seedlings. (c) Dry weight of seedlings. (d) Soluble sugar content of seedlings. (e) Chlorophyll content of seedlings. (f) Glucose content of seedlings. (g) Protein content of seedlings. (h) P content of seedlings. (i) Activity of roots of the seedlings. Data and error bars are the mean ± SE (n = 6), and “n.s.” indicates no significant differences (unpaired two-tailed Student’ s *t*-test, **p* > 0.05).


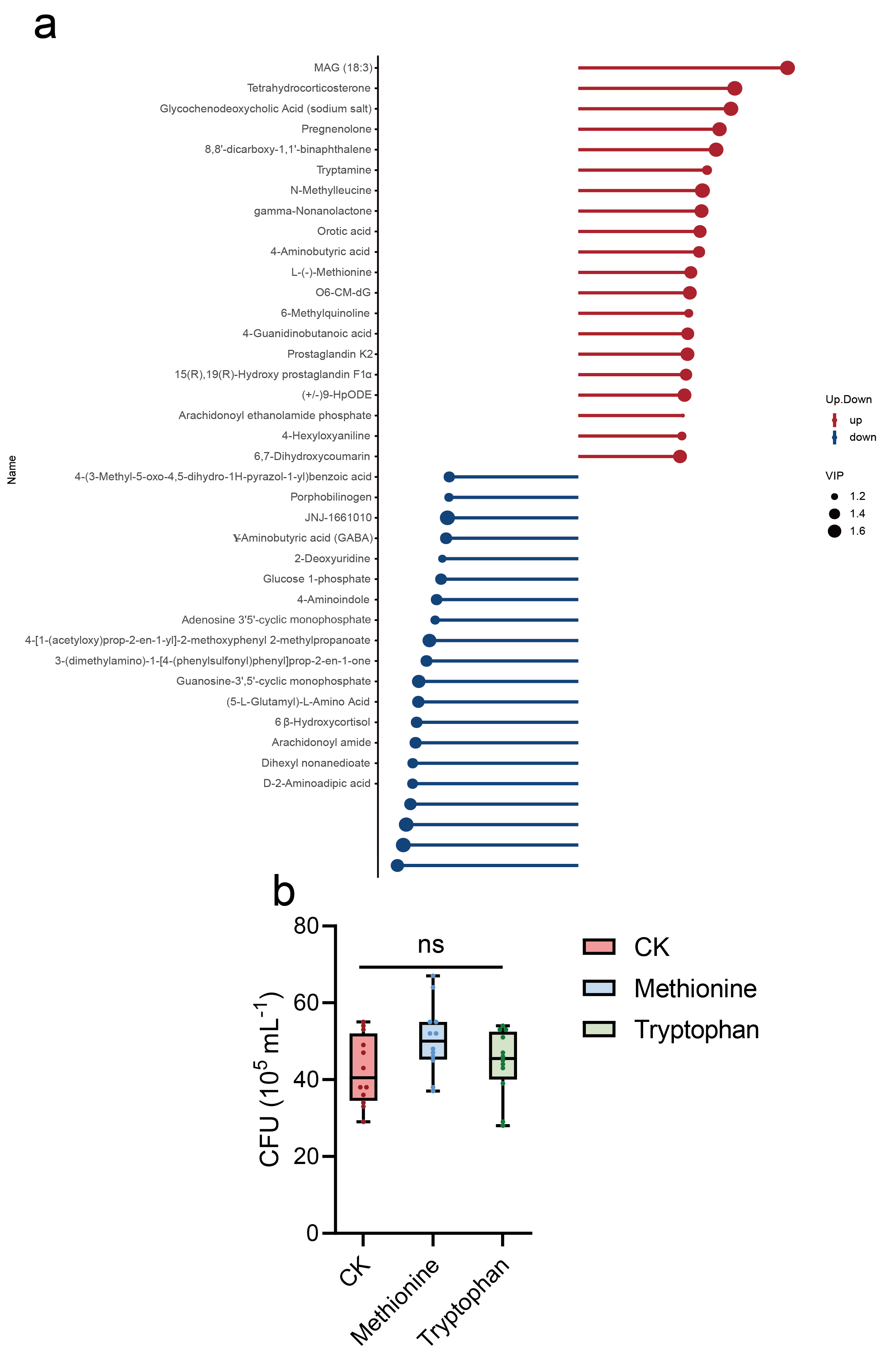


**Fig. S11. Analysis of mycelial exudates and their effects on bacterial chemotaxis.** (a) Metabolic profiling of mycelial exudates from *S. clintonianus* compared to *C. lacerata*. (b) Chemotactic response of *B. altitudinis* B4 to selected amino acids. Data and error bars are the mean ± SE (n = 12), and “n.s.” indicates no significant differences (One-way analysis of variance with Tukey’s test, *p* > 0.05).

Table S1 Primers used in this study

| Gene name/ description | | | Sequences | | | | |
| --- | --- | --- | --- | --- | --- | --- | --- |
| ITS1-F | | | CTTGGTCATTTAGAGGAAGTAA | | | | |
| ITS4-R | | | TCCTCCGCTTATTGATATGC | | | | |
| 16S rRNA 27-F | | | AGAGTTTGATCCTGGCTCAG | | | | |
| 16S rRNA 1492-R | | | CTACGGCTACCTTGTTACGA | | | | |
| thiD-F | | | GGTGGAGCAGGTATTCAA | | | | |
| thiD-R | | | GAGTGCCTCGATAGACAGA | | | | |
| thiG-F | | | CCAGGTGCTTCTCCAATC | | | | |
| thiG-R | | | CCTACACCCGCATCAATA | | | | |
| tenA-F | | | TGAAACAAACAACGCCTGAC | | | | |
| tenA-R | | | CTCCCAGAAATGGTATTCGTAA | | | | |
| thiC-F | | | AAAGTGAATGGAATCGCAGA | | | | |
| thiC-R | | | TACCTTAACAGGACGCCCGC | | | | |
| THI73-F | | | CCCTGACTGACCCTAA | | | | |
| THI73-R | | | GCTGAATCCGAACGAA | | | | |
| thiE-F | | | GTTGGGATGGGTCCAGTCTA | | | | |
| thiE-R | | | GGTGCCGCATTTTCATATGT | | | | |
| DXS-F | | | AAGGTAAATCAATCCGTGTT | | | | |
| DXS-R | | | CTGTGTTTTCGTCAGTCCAA | | | | |
| thiL-F | | | TGATGTGAGTGACGGATTAG | | | | |
| thiL-R | | | CGGTAGATGTGGATGTTTTG | | | | |
| *S. clintonianus*-F | | | TTACAACTTTCAGCAA | | | | |
| *S. clintonianus*-F | | | ACTATCCAAGCACCCT | | | | |
| motA-F | | | CGGAGAGACATCAGTCAGGC | | | | |
| motA-R | | | AAGTGCGGCAATAAGTCCGA | | | | |
| motB-F | | | CTCGCCCTCTTCATTGTG | | | | |
| motB-R | | | TTTCTGTTTCCTCGGTGC | | | | |
| filG-F | | | AATGATTTCCTTAGGGCTAG | | | | |
| filG-R | | | GCGATGGCAATACTGTGA | | | | |
| MCP-F | | | AGAAGGCCGTGAGCAAATGA | | | | |
| MCP-R | | | GCCGCATGTTCTTCTGACAC | | | | |
| β-actin (reference gene) | | | GCTACGTTGGTGATGAGGCT | | | | |
|  |  |  | AACACGCAGCTCGTTGTAGA | | | | |
| 16S rRNA (reference gene) | | | GGTCCCCCTCTTTGGTCTTG | | | | |
|  |  |  | CGGGTGAGTAATGTCTGGGG | | | | |
| **Table S2 The original physical and chemical properties of the soil** | | | | | | | |
| TN (g kg^-1^) | TK (g kg^-1^) | N (mg kg^-1^) | | P (mg kg^-1^) | K (mg kg^-1^) | PH | SOM(%) |
| 0.38±0.05 | 14.12±2.58 | 49.53±8.75 | | 39.39±1.34 | 90.95±6.87 | 5.73±0.09 | 0.24±0.02 |

**Methods for supplementary materials**

**Effects of *B. altitudinis* B4 organic volatiles on *S. clintonianus***

A two-compartment plate system was employed to assess the interaction between *S. clintonianus* and *B. altitudinis* B4 organic volatiles. Each plate contained P20 medium (without thiamine) to standardize nutrient conditions. A fungal plug (8 mm) of *S. clintonianus*, taken from growing edge of a pre-cultured colony, was aseptically transferred to the center of one compartment. The *B. altitudinis* B4 (5 μL, OD_600_ = 0.4) was inoculated at the opposing compartment. 5 μL of sterile water was used as control. The plates were incubated at 28°C under dark conditions. Image J was used for measuring the area of mycelium after 14 days.

**Dispersal of *B. altitudinis* B4 on fungal hyphae**

To determine whether *B. altitudinis* B4 is attracted by *S. clintonianus* and moves along fungal hyphae, synthetic fungal hyphae, made of sterile glass fibers (8 μm, immersion in sterile ddH_2_O or mycelial exudates of *S. clintonianus*) were created on the surface of P20 ^[1]^. A 1-μl spot of *B. altitudinis* B4 (OD_600_ = 0.4) was inoculated at the center of each plate. After 24 hours of growth, *B. altitudinis* B4 dispersal on the glass fibers was observed at a distance of 1 cm from the center of the colony. The experiment was conducted in three independent replicates, and representative photographs of the plates were taken.

***S. clintonianus* exudates collection in the combined system**

*Suillus clintonianus* fungal plugs and *B. altitudinis* B4 were cultured in P20 liquid medium (without thiamine) at 28°C for 14 d, then the plugs were washed twice with sterile water to remove residual medium, and transferred to 25 mL of sterile water. After 24 h, the mycelium and hyphae were removed and mycelial secretion was collected and freeze-dried after filtering through a 0.22-μm membrane (Merck Millipore, Germany) to obtain the fungal mycelial secretion in the combined system.

**Effects of *S. clintonianus* exudates on *B. altitudinis* B4 in the low carbon environment**

To simulate a low-carbon environment for *S. clintonianus*, the glucose concentration in the P20 medium was adjusted from the standard cultivation level (10 g L⁻¹) to reduced concentrations of 7.5, 5, and 2.5 g L⁻¹. To collect hyphal secretions, *S. clintonianus* was cultured in the aforementioned medium at 28°C for 14 days. The mycelia were then rinsed twice with sterile ddH_2_O to remove residual medium components. The mycelial secretion was collected after 24 h and freeze-dried after filtering through a 0.22-μm membrane (Merck Millipore, Germany). Subsequently, the aforementioned mycelium secretions were used to conduct capillary chemotaxis experiments, with sterile water serving as the control ^[2]^.

**Determination of bacterial chemotaxis-related genes**

The *B. altitudinis* B4 (1 μL, OD_600_ = 0.4) was inoculated in swimming medium (1% tryptone, 0.5% NaCl, 0.25% glucose, 0.3% agar) at 30°C for 8 h. Bacterial RNA was extracted from the medium with a TRIzolR reagent according to the instructions (Invitrogen, USA). Only high-quality RNA samples (OD_260/280_= 1.8-2.2, OD_260/230_ ≥ 2.0, RIN ≥ 8.0, 28S:18S ≥ 1.0, ≥ 1 μg) were selected to obtain cDNA by reverse transcription according to the kit. cDNA was obtained by reverse transcription using the AceQ qPCR SYBRRGreen Master Mix (Vazyme Biotech Co., Ltd, China) on a 7500 Real-Time PCR system (Applied Biosystems), and the PCR conditions were 95°C for 5 min, 95°C for 10 s, 60°C for 30 s, and 72°C for 20 s, with a total of 40 cycles. The selected genes (*motA*, *motB*, *filG*, and *MCP*) and the primers are listed in Supplementary Table S1 ^[3]^. The internal reference gene was selected as *B. altitudinis* B4 *gyrB* gene, and the expression of the selected genes was finally normalized by calculating the relative expression by the 2^-ΔΔCt^ method ^[4]^.

**Fungal hyphae-mediated bacterial transport and its direct effects on plant growth**

*P. massoniana* seeds were surface-sterilized with 70% (v/v) ethanol and 3% (v/v) sodium hypochlorite for 3 min, then washed 3 times with sterile water. The surface-sterilized seeds were germinated on sterilized vermiculite at 28°C in the dark. The seedlings were transplanted to the plant compartment after 30 days. *B. altitudinis* B4 suspension were added onto the roots of *P. massoniana* seedlings at a final bacterial concentration of OD_600_ = 0.0005 ^[5]^. The growth index of the *P. massoniana* seedlings was measured at 90 days post-inoculation, including plant height, root length, dry weight, chlorophyll content, root activity, soluble sugar content, glucose content, protein content and the nitrogen (N) and phosphorus (P) contents ^[6, 7, 8]^.

**Soil DNA extraction and quantitative RT-PCR assays**

Soil from potted plants was collected, including *S. clintonianus*-only treatment (Sc) and combined treatment (Sc+B4), and total soil DNA was extracted from the root zone and mycelial zone using soil DNA extraction reagent. The qPCR program was set as follows: 95°C for 5 min; 40 cycles of 95°C for 10 s, 60°C for 30 s, and 72°C for 20 s. A melting curve was recorded at the end of each run to verify the specificity of the reaction. The *S. clintonianus* β-actin gene was used as reference genes to normalize the expression of selected genes using the 2^-△△Ct^ method ^[4]^. The experiment was performed with three individual replicates and three technical replicates.

**Supplementary References:**

1. Zhang YC, Kastman EK, Guasto JS, et al. Fungal networks shape dynamics of bacterial dispersal and community assembly in cheese rind microbiomes. Nat Commun. 2018;9:336. https://doi.org/10.1038/s41467-017-02522-z
2. Caetano-Anolles G, Crist-Estes DK, Bauer WD. The chemotaxis of *Rhizobium meliloti* to the plant flavone luteolin requires functional nodulation genes. J Bacteriol. 1988;170(7):3164-69. <https://doi.org/10.1128/jb.170.7.3164-3169.1988>
3. Mei Y, Zhang ML, Cao GY, et al. Endofungal bacteria and ectomycorrhizal fungi synergistically promote the absorption of organic phosphorus. Plant Cell Environ. 2024;47(2):600-10. <https://doi.org/10.1111/pce.14742>
4. Ma KW, Niu YL, Jia Y, et al. Coordination of microbe-host homeostasis by crosstalk with plant innate immunity. Nat Plants. 2021;7(6):814-25. https://doi.org/10.1038/s41477-021-00920-2
5. Schmittgen TD, Livak KJ. Analyzing real-time PCR data by the comparative CT method. Nat Protoc. 2008;3(6):1101-8. https://doi.org/10.1038/nprot.2008.73
6. Liu J, Ni J, Mo AL, et al. Cadmium affects the growth, antioxidant capacity, chlorophyll content, and homeostasis of essential elements in soybean plants. S Afr J Bot. 2023;162:604-10. https://doi.org/[10.1016/j.sajb.2023.09.059](http://dx.doi.org/10.1016/j.sajb.2023.09.059)
7. Warner MH, Jones Jr JB. A rapid method for nitrogen determination in plant tissue. Commun Soil Sci Plan. 1970;1(2):109-14. <https://doi.org/10.1080/00103627009366247>
8. Pansu M, Gautheyrou J. Handbook of soil analysis: mineralogical, organic and inorganic methods. Berlin, Heidelberg, New York: Springer. 2006.
